# Downregulation of Chloroplast RPS1 Negatively Modulates Nuclear Heat-Responsive Expression of *HsfA2* and Its Target Genes in *Arabidopsis*

**DOI:** 10.1371/journal.pgen.1002669

**Published:** 2012-05-03

**Authors:** Hai-Dong Yu, Xiao-Fei Yang, Si-Ting Chen, Yu-Ting Wang, Ji-Kai Li, Qi Shen, Xun-Liang Liu, Fang-Qing Guo

**Affiliations:** The National Key Laboratory of Plant Molecular Genetics and National Center for Plant Gene Research (Shanghai), Institute of Plant Physiology and Ecology, Shanghai Institutes for Biological Sciences, Chinese Academy of Sciences, Shanghai, China; Peking University, China

## Abstract

Heat stress commonly leads to inhibition of photosynthesis in higher plants. The transcriptional induction of heat stress-responsive genes represents the first line of inducible defense against imbalances in cellular homeostasis. Although heat stress transcription factor *HsfA2* and its downstream target genes are well studied, the regulatory mechanisms by which *HsfA2* is activated in response to heat stress remain elusive. Here, we show that chloroplast ribosomal protein S1 (RPS1) is a heat-responsive protein and functions in protein biosynthesis in chloroplast. Knockdown of *RPS1* expression in the *rps1* mutant nearly eliminates the heat stress-activated expression of *HsfA2* and its target genes, leading to a considerable loss of heat tolerance. We further confirm the relationship existed between the downregulation of *RPS1* expression and the loss of heat tolerance by generating RNA interference-transgenic lines of *RPS1*. Consistent with the notion that the inhibited activation of *HsfA2* in response to heat stress in the *rps1* mutant causes heat-susceptibility, we further demonstrate that overexpression of *HsfA2* with a viral promoter leads to constitutive expressions of its target genes in the *rps1* mutant, which is sufficient to reestablish lost heat tolerance and recovers heat-susceptible thylakoid stability to wild-type levels. Our findings reveal a heat-responsive retrograde pathway in which chloroplast translation capacity is a critical factor in heat-responsive activation of *HsfA2* and its target genes required for cellular homeostasis under heat stress. Thus, RPS1 is an essential yet previously unknown determinant involved in retrograde activation of heat stress responses in higher plants.

## Author Summary

As a consequence of global warming, increasing temperature is a serious threat to crop production worldwide and may influence the objectives of breeding programs. As a universal cellular response to a shift up in temperature, the heat stress response represents the first line of inducible defense against imbalances in cellular homeostasis in the prokaryotic and eukaryotic kingdoms. Given that components of the photosynthetic apparatus housed in the chloroplast are the primary susceptible targets of thermal damage in plants, the chloroplasts were proposed as sensors to a shift up in temperature. However, the mechanism by which chloroplasts regulate the expression of nuclear heat stress–responsive gene expression according to the functional state of chloroplasts under heat stress remains unknown. In this study, we have identified chloroplast ribosomal protein S1 (RPS1) as a heat-responsive protein through proteomic screening of heat-responsive proteins. We have established a previously unrecognized molecular connection between the downregulation of *RPS1* expression in chloroplast and the activation of *HsfA2*-dependent heat-responsive genes in nucleus, which is required for heat tolerance in higher plants. Our data provide new insights into the mechanisms whereby plant cells modulate nuclear gene expression to keep accordance with the current status of chloroplasts in response to heat stress.

## Introduction

It is generally accepted that a temperature upshift, usually 10–15°C above an optimum temperature for growth, is considered as heat stress for leaf photosynthesis in higher plants [1], [2]. Photosystem II (PSII) is the most heat sensitive apparatus within the chloroplast thylakoid membrane protein complexes involved in photosynthetic electron transfer and ATP synthesis [1]–[5]. Chlorophyll fluorescence, the ratio of variable fluorescence to maximum fluorescence (*Fv/Fm*) and the base fluorescence (*F*_o_) are used as common indicators of heat stress-induced damages that have been shown to correlate with alterations of photochemical reactions in thylakoid lamellae of chloroplast [1], [2], [6], [7]. Oxygen evolving complex (OEC) in PSII is highly thermolabile and heat stress may cause the dissociation of OEC, resulting in an imbalance in the electron flow from OEC toward the acceptor side of PSII in the direction of PSI reaction center [1]–[3], [8], [9]. Studies on spinach thylakoids subjected to heat stress have shown that heat stress causes cleavage of the reaction center-binding protein D1 of PSII and induces dissociation of a manganese (Mn)-stabilizing 33-kDa proteins from PSII reaction center complex [10]. Besides the disruption of OEC in PSII, heat stress also leads to dysfunction in the system of carbon assimilation metabolism in the stroma of chloroplast [5]. The rate of ribulose-1,5-bisphosphate (RuBP) regeneration is limited by the disruption of electron transport and inactivation of the oxygen evolving enzymes of PSII [7], [11]. It is known that under heat stress, the decline in ribulose-1,5-bisphosphate carboxylase/oxygenase (Rubisco) activity is mainly due to inactivation of Rubisco activase that is extremely sensitive to elevated temperatures because the enzyme Rubisco of higher plants is heat stable [5], [11]. In addition to the early effects on photochemical reactions and carbon assimilation, heat stress usually leads to alterations in the microscopic ultrastructures of chloroplast and the integrity of thylakoid membranes, including membrane destacking and reorganization [2], [12]–[15].

Because temperature elevations represent a fundamental challenge to all sessile organisms, higher plants are capable of a variety of heat shock responses characterized by a rapid expression reprogramming of a set of proteins known as heat shock proteins (HSPs) [16], [17]. Analysis of Arabidopsis genome-wide expression profiles to heat stress has shown that the transcripts of the well-characterized *HSPs* increased dramatically, including *Hsp101*, *Hsp70s* and small *HSPs*, which are proposed to act as molecular chaperones in protein quality control under heat stress [18]–[23]. In addition to classical heat stress responsive genes, these studies have also revealed the involvement of factors in heat tolerance, including members of the dehydration-responsive element-binding transcription factor 2 (DREB2) family of transcription factors, *GALACTINOL SYNTHASE 1* (*GolS1*) in the raffinose oligosaccharide (RFO) pathway, and *ASCORBATE PERROXIDASE2* (*APX2*) [18]–[27]. The accumulation of HSPs is assumed to counteract the detrimental effects of protein misfolding and aggregation that result from heat stress [28], [29]. Genetic analyses demonstrate that Hsp101 is required for heat tolerance, functioning in cooperation with the small HSPs to resolubilize protein aggregates after heat stress in higher plants [30], [31]. Small HSPs may function as membrane stabilizers and possibly as site-specific antioxidants to protect thylakoid membranes against heat stress [2], [32]. Several small HSPs have been reported to protect thylakoid stability from heat or oxidative stresses in photosynthetic organisms such as higher plants [33]–[35] and cyanobacteria [36], [37].

Expression of *HSP* genes is orchestrated mainly at the transcriptional level by heat shock transcription factors (HSFs) that recognize *cis*-elements (heat shock elements; HSEs) conserved in *HSP* gene promoters [16], [17]. HSFs play a central role in heat shock response in many species. In contrast to *Drosophila*, *Caenorhabditis elegans* and yeast that have a single HSF, the Arabidopsis genome contains 21 *HSFs* that are assigned to 3 classes, A, B and C, based on the structural features of their oligomerization domains [38]. At least 23 and 18 *HSF* genes were identified in rice (*Oryza sativa*) and tomato (*Lycopersicon esculentum*), respectively [39], [40]. In comparison with class A HSFs, the members of class B lack the structural motif (aromatic, hydrophobic and acidic amino acids) present in the C-terminal domain crucial for the activator activity of class A HSFs [41]. In agreement with the structural difference between class A and B HSFs, the transient overexpression of several class B HSFs failed to activate heat shock responsive promoters in tobacco protoplasts [42], [43], indicating that certain class B HSFs may function as coactivators to enhance transcriptional levels of house-keeping genes during heat stress. Recently, consistent with having repressive activities [44], Arabidopsis HsfB1 and HsfB2b were reported to act as repressors that negatively regulate the expression of *HSFs*, including *HsfA2*, in response to heat stress, indicating that these two B class members may interact with class A HSFs in regulating the shut-off of the heat shock response [45], [46].

As one of the most intensely studied HSFs, HsfA2 is considered as a key regulator of heat tolerance in tomato [41], [47], [48] and *Arabidopsis* [21], [49], [50] owing to its high activator potential for transcription of *HSP* genes and its continued accumulation during repeated cycles of heat stress and recovery [17]. In tomato, HsfA1a, a constitutively expressed HSF, regulates the transcriptional activation of *HsfA2* and *HsfB1* in response to heat stress, indicating that these three HSFs seem to form a regulatory network to regulate the expression of down-stream heat shock-responsive genes [40], [51]. In contrast to tomato, Arabidopsis HsfA2 as a transcriptional activator can localize to the nucleus and is regulated by a complex “master switch” containing HsfA1a-e [52]–[54]. Interestingly, Arabidopsis ROF1 (AtFKBP62, a peptidyl prolyl cis/trans isomerase) also modulates thermotolerance by interacting with HSP90.1 and affecting the accumulation of HsfA2-regulated sHSPs [55]. The major target genes regulated by HsfA2 in *Arabidopsis* have been identified by analyzing *HsfA2* knockout mutant and overexpression transgenic plants [21], [49]. These target genes encode APX2, GolS1, several small Hsps and individual isoforms of the Hsp70 and Hsp101 families. In addition to the induction by heat stress, the expression levels of *HsfA2* were also up-regulated in response to high light and H_2_O_2_ [49]. Interestingly, a recent report has shown that sumoylation of HsfA2 by the small ubiquitin-like modifier protein (SUMO) regulates its activity in connection with heat stress response and heat tolerance in *Arabidopsis* [56].

Although the accumulating literatures on the functions of HSPs and HSFs have substantially extended our understanding of heat stress response in plants, its regulatory network is far from completely understood. In this study, we have identified chloroplast ribosomal protein S1 (RPS1) as a heat-responsive protein through proteomic screening of heat-responsive proteins. In *Escherichia coli*, RPS1, the largest ribosomal protein, is involved in the process of mRNA recognition and binding by the 30S ribosomal subunit to the translation initiation site [57]–[59]. The RPS1 in *E. coli* consists of six repetitions of a conserved structural domain, called S1 domain, which is found in many other proteins involved in RNA metabolism in all organisms [57], [58]. In bacteria, RPS1 is believed to facilitate the binding of the 30S small ribosomal subunit near the initiation codon of the transcripts [59], [60]. A homologue of the bacterial S1 protein was found in spinach chloroplast [61], [62], cyanobacteria [63] and *Chlamydomonas reinhardtii* [64], [65].

With the identification of RPS1 as a heat-responsive protein, we have further demonstrated that knockdown of *RPS1* expression leads to inhibition of transcriptional activation of *HsfA2* and its target genes in the *rps1* mutant, which confers a heat-sensitive phenotype. Furthermore, our findings support that the capacity of plastid protein translation is critical for retrograde activation of *HsfA2*-dependent heat tolerance pathway. Our findings shed new light on the mechanisms whereby plant cells modulate nuclear gene expression to keep accordance with the current status of chloroplasts in response to heat stress.

## Results

### RPS1 Is a Heat-Responsive Protein Required for Heat Tolerance

**Figure 1:**
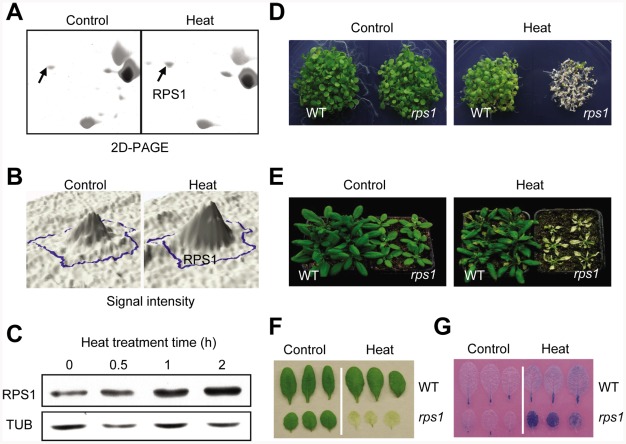
RPS1 is a heat-responsive protein required for heat tolerance. (A) Zoom-in view of the RPS1 spot in the images of 2D-PAGE electrophoresis separation of proteins in response to heat treatment (38°C, 2 h) in dark. The fully-expanded leaves were detached from 21-d-old wild type (WT) plants for control or heat treatment. (B) The quantified silver-staining signal intensity of the RPS1 spot according to the images shown in (A). (C) Western blot analysis showing RPS1 protein levels in wild type leaves in response to heat treatment (38°C) in dark for the indicated time with an anti-RPS1 polyclonal antibody. Equal protein loading was confirmed with antiserum against α-Tubulin. (D) to (F) The *rps1* mutant plants showing heat-sensitive phenotypes compared with WT plants as examined with young seedling (D), whole plant (E) and detached leaf (F). Heat treatments were performed as described in Materials and Methods. (G) Trypan blue staining of the heat-challenged detached leaves of WT and the *rps1* plants as described in (F).

**Figure 2:**
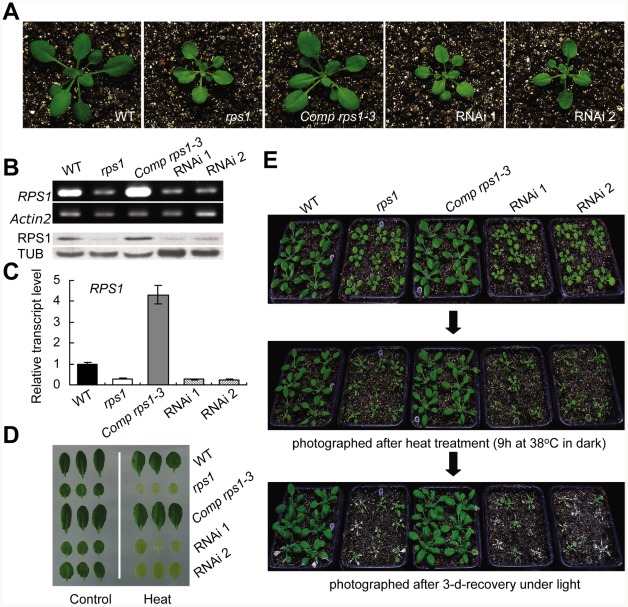
*RPS1*–RNA interference leads to defects in heat tolerance. (A) Phenotypes of WT, *rps1*, *rps1* complemented with *RPS1* genomic DNA, and two *RPS1*-RNA interference lines RNAi 1 and 2 as 21-d-old plants. (B) to (C) RT-PCR, western blot and qRT-PCR analysis showing expression and translation levels of *RPS1* in leaves excised from different genotypes as described in (A). For qRT-PCR analysis, *Actin2* was used as the internal standard. Error bars indicate standard deviations of three technical replicates, and the results were consistent in three biological replicates. (D) to (E) Heat-sensitive phenotypes of different genotypes as described in (A) as examined with detached leaf and whole plant assays performed as described in Materials and Methods.

The objective of this study was the identification of novel genes and pathways that contributed to the regulatory networks involved in the development of heat tolerance in *Arabidopsis*. During our initial studies, we were particularly interested in the responsive proteins that are predicted to be targeted to chloroplast since photosynthesis housed in chloroplast is extremely sensitive to heat stress [1], [2]. To uncover how plant cells modulate protein expression in response to heat stress, we performed a proteomic screen for heat-responsive proteins. One heat-responsive protein was identified as the chloroplast RPS1 (Figure 1A, 1B, Figures S1 and S2), equivalent to the plastid ribosomal protein S1 orthologues CS1 in spinach [61], [62] and CreS1 in *C. reinhardtii* [65] (Figure S3), which have been reported to specifically bind chloroplast mRNA during translation initiation. Western blot analysis further confirmed that the protein levels of RPS1 gradually increased to peak at 2 h after heat treatment, indicating that RPS1 is a heat-inducible protein (Figure 1C and Figure S4). Interestingly, RPS1 responded to heat stress at protein level, but not at transcriptional level since the expression levels of *RPS1* slightly decreased during 2-h heat treatment (Figure S4), suggesting that RPS1 may not be identified as a heat-responsive protein through the analysis of heat-responsive transcriptome because the correlation between mRNA and protein levels is not sufficient to predict protein expression levels from the quantitative mRNA data [66], [67]. Given that RPS1 was induced by heat (Figure 1A–1C and Figure S4) and its highly conserved orthologues are involved in the direct control of chloroplast gene expression, we reasoned that RPS1 might act as a retrograde communication coordinator to trigger nuclear gene expression critical for heat tolerance. We first examined whether RPS1 plays a role in heat tolerance by identifying a homozygous knockdown mutant of *rps1* with a T-DNA insertion at 6 bp from the 5′-untranslated region (5′-UTR) of the *RPS1* gene (Figure 2A and Figure S5). The substantial reduction in *RPS1* expression in the *rps1* mutant compared with wild type was verified using RT-PCR, western blots and quantitative PCR with reverse transcription (qRT-PCR) (Figure 2B and 2C). To analyze *RPS1* expression, we generated transgenic Arabidopsis plants carrying *pRPS1:GUS* constructs and the GUS staining signals were highly observed in cotyledons and true leaves (Figure S6). The *rps1* mutant plants appeared slightly pale green with a reduced plant size (Figure 2A). When 2.5-d-old seedlings were exposed to transient increases in temperature, almost none of the mutant seedlings survived after a 7-d recovery, compared with a survival rate of greater than 90% for wild type seedlings (Figure 1D). In addition, both mature plants and detached leaves of the *rps1* mutant exhibited heat-sensitive phenotypes compared with wild type plants after heat treatment and recovery (Figure 1E and 1F). Cell death was examined by Trypan blue staining in the detached leaves challenged with heat stress shown in Figure 1F, and the cell death phenotype of the *rps1* mutant was considerably more severe than that of wild type (Figure 1G). Given the diminished expression of *RPS1* in the *rps1* mutant, these results indicate that *RPS1* is required for heat tolerance. To test whether *RPS1* is generally involved in abiotic stress responses, we monitored the sensitivity of wild type and *rps1* mutant seedlings to salt and osmotic stresses and observed no significant difference under either treatment between wild type and *rps1* mutant plants (Figures S7 and S8). These data support the assumption that the alteration in *RPS1* expression affects cellular heat stress response by disrupting specific machinery rather than through general physiological defects.

To further confirm the relationship existed between the downregulation of *RPS1* expression and the loss of heat tolerance observed in the *rps1* mutant, we investigated whether RNA interference (RNAi)-mediated gene silencing of *RPS1* alters the heat-responsive behavior of transgenic plants harboring RNAi constructs. As expected, we found that the RNAi lines, in which downregulation of *RPS1* expression was validated using RT-PCR, western blots and qRT-PCR (Figure 2B and 2C), appeared pale green like those of the *rps1* mutant (Figure 2A) and exhibited a heat sensitive phenotype in detached leaves and whole plants in comparison with wild type plants (Figure 2D and 2E). Furthermore, transforming genomic fragments of *RPS1* complemented the defects of the *rps1* mutant in heat tolerance (Figure 2D and 2E). These results demonstrate that *RPS1* is the gene responsible for the deficiency in heat tolerance exhibited in the *rps1* mutant. In addition, three genomic complementation lines (*Comp rps1-2*, *1-3* and *1-4*) were generated in which the expression level of *RPS1* dramatically increased when compared with wild type (Figure 2B, 2C and Figure S9). To test if the increased expression levels of *RPS1* could enhance the heat tolerance, we performed the heat tolerance assay using the seedlings of wild type and the complementation lines. The result showed that when 2.5-d-old seedlings were exposed to transient increases in temperature, almost none of the wild type seedlings survived after a 7-d recovery, compared with a survival rate of 40–50% for the complementation line seedlings (Figure S9). On the other hand, we also analyzed the translation efficiency of the representative thylakoid membrane proteins encoded by chloroplast DNA, including D1, D2, CP43, CP47, PsaA, PsaB and β-subunits of ATPase between wild type and the genomic complementation line *Comp rps1-3* in which the transcript level and protein level of *RPS1* increased dramatically compared with wild type (Figure 2B and 2C). In agreement with the enhancement in heat-tolerance, the translation efficiency of the representative thylakoid membrane proteins in complementation line *Com rps1-3* increased substantially in comparison with the wild type seedlings under both control and heat stress conditions (Figure S9C). These data further support that RPS1 is required for heat tolerance.

### Knockdown of *RPS1* Expression Inhibits Activation of *HsfA2*-Dependent Heat Stress Responses

**Figure 3:**
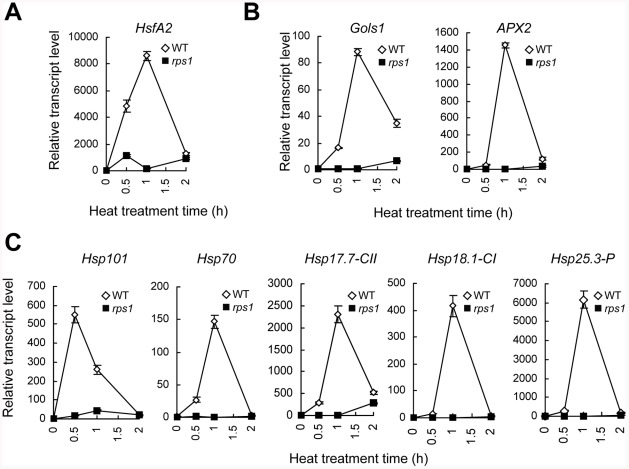
Downregulation of *RPS1* expression inhibits transcriptional activation of *HsfA2* and its target genes in response to heat stress. qRT-PCR analysis of mRNA levels of *HsfA2* (A), its representative target genes, including *GolS1*, *APX2* (B) and *HSP* genes (C) in detached, fully-extended WT and *rps1* leaves challenged with heat treatment (38°C) for the indicated time in dark. *Actin2* was used as the internal standard. Error bars indicate standard deviations of three technical replicates, and the results were consistent in three biological replicates.

Based on heat-responsive transcriptional analysis, *HsfA2* is the most inducible *HSF* gene and appears to play a key role not only in the triggering of cellular responses to heat stress, but also in the amplification of the signal in the responses [49], [50]. *HsfA2* knockout mutant displays a heat-sensitive phenotype [50], indicating that *HsfA2* is a key heat tolerance regulator that cannot be replaced by other *HSF* genes. We verified the expression pattern of *HsfA2* in the *rps1* mutant in response to heat by qRT-PCR. At a heat stress temperature (38°C), the levels of *HsfA2* mRNA rapidly increased and peaked 1 h after treatment in wild type plants whereas the heat-activated expression of *HsfA2* was severely inhibited in the *rps1* mutant (Figure 3A). In agreement with the *HsfA2* expression pattern, the heat-responsive expression of a subset of representative *HsfA2* target genes, including *APX2*, *GolS1* and several *HSP*s (*Hsp17.7-CII*, *Hsp18.1-CI*, *Hsp25.3-P*, *Hsp70* and *Hsp101*) [49], was nearly abolished in the *rps1* mutant and did not match the transcriptional activity of these genes in wild type plants (Figure 3B and 3C). In addition, we examined the heat-responsive expression levels of 15 members in class A *HSF* by qRT-PCR analysis. In agreement with the previous reports [19], [49], we found that *HsfA2* is the most inducible *HSF* gene among 15 members in class A and the mutation of *RPS1* leads to the most pronounced inhibitory effect on heat-responsive transcriptional activation of *HsfA2* in comparison with the rest of the class A members whose relative expression levels are less than 100 with no or marginal difference between wild type and the *rps1* mutant after heat treatment (Figure S10). These results indicate that downregulation of *RPS1* expression considerably inhibits the transcriptional activation of *HsfA2* and its target genes in response to heat stress, which are required for establishing cellular heat tolerance.

### Constitutive Expression of *HsfA2* Is Sufficient to Reestablish Lost Heat Tolerance in *rps1* Mutant Plants

**Figure 4:**
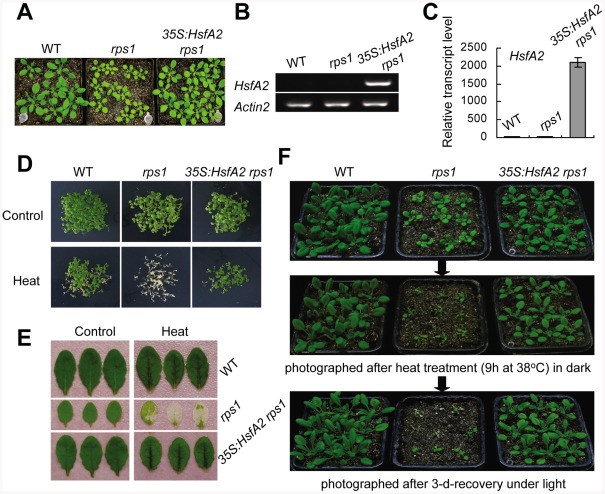
Overexpression of *HsfA2* complements the heat-sensitive phenotype of *rps1* mutant plants. (A) Phenotypes of WT, *rps1* and *rps1* with a *35S:HsfA2* cDNA transgene as 21-d-old plants. (B) to (C) *HsfA2* mRNA levels in leaves of the plants described in (A) were analyzed by RT-PCR (B) and qRT-PCR (C). *Actin2* was used as the internal standard. Error bars indicate standard deviations of three technical replicates, and the results were consistent in three biological replicates. (D) to (F) Heat-challenged phenotypes of different genotypes as described in (A) as examined with young seedling (D), detached leaf (E) and whole plant (F) assays performed as described in Materials and Methods.

**Figure 5:**
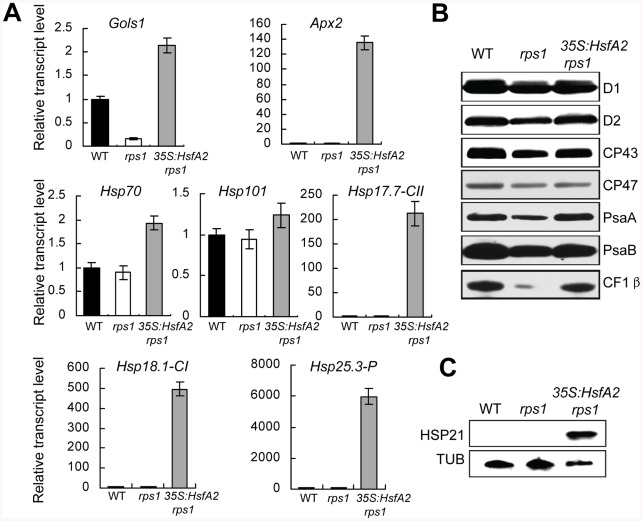
Overexpression of *HsfA2* leads to constitutive expression of its target genes in *rps1* mutant plants. (A) qRT-PCR analysis of mRNA levels of the representative *HsfA2* target genes in the fully-extended leaves of WT, *rps1* and *rps1* with a *35S:HsfA2* cDNA transgene plants. *Actin2* was used as the internal standard. Error bars indicate standard deviations of three technical replicates, and the results were consistent in three biological replicates. (B) Western blot analysis of thylakoid membrane proteins extracted from the leaves of the genotypes indicated in (A). Equal protein loading was determined by contents (2 µg) of chlorophyll in thylakoid membrane extracts according to (Peng et al., 2006). (C) Protein levels of HSP21, encoded by *HsfA2* target gene *Hsp25.3*, in leaves of the plants described in (A) were analyzed by western blots with a polyclonal antibody against HSP21.

Many of *HsfA2* target genes are involved in protective environmental stress responses, including *APX2* encoding a enzyme scavenging stress-induced reactive oxygen species (ROS) [68], *GolS1* encoding a enzyme catalyzing the synthesis of protective osmolyte such as RFO [69] and several *HSPs* encoding chaperone proteins stabilizing damaged proteins [49]. In this view, the defects in heat tolerance observed in the *rps1* mutant are likely to be due to the repressed expression of *HsfA2* and its target genes in response to heat stress. To further define the role of *HsfA2* as a mediator in the activation of RPS1-dependent heat-responsive processes, we generated transgenic *rps1* plants with the *HsfA2* gene under the control of constitutive CaMV35S promoter (Figure 4A). The constitutive expression of *HsfA2* in the *35S:HsfA2 rps1* mutant was verified using RT-PCR and qRT-PCR (Figure 4B and 4C). Notably, constitutive expression of *HsfA2* almost totally restored the heat-sensitive phenotype of the *rps1* mutant compared with wild type in young seedlings, detached leaves and whole plants (Figure 4D–4F). Furthermore, we have performed qRT-PCR and western blot analysis to examine the expression levels of *HsfA2* target genes and the protein levels of thylakoid membrane proteins in *35S:HsfA2 rps1* plants, respectively. The results showed that the expression levels of a subset of representative *HsfA2* target genes, including *APX2*, *GolS1* and several *HSP*s (*Hsp17.7-CII*, *Hsp18.1-CI*, *Hsp25.3-P*, *Hsp70* and *Hsp101*), constitutively increased in *35S:HsfA2 rps1* plants compared with that in wild type and *rps1* plants (Figure 5A). These results further confirm that the reduced expression levels of *HsfA2* and its target genes under heat stress are responsible for the heat-sensitive phenotype of the *rps1* mutant. Previous studies indicate that HSP21 is targeted to chloroplast and functions in protecting chloroplasts from heat or oxidative stresses in higher plants [34], [35]. Importantly, by performing western blot analysis, we further confirmed that the protein level of HSP21, encoded by the *HsfA2* target gene *Hsp25.3-P*, also increased dramatically in *35S:HsfA2 rps1* plants in comparison with WT and the *rps1* mutant plants (Figure 5C). On the other hand, we found that the protein levels of thylakoid membrane proteins represented by D1, D2, CP43, CP47, PsaA, PsaB and β-subunits of ATPase in *35S:HsfA2 rps1* plants substantially increased in comparison with the *rps1* mutant, and were restored to near the wild type levels (Figure 5B). These data reveal that HsfA2 acts downstream of RPS1 and plays an essential role in mediating chloroplast RPS1-initiated transcriptional reprogramming of downstream target genes critical for heat tolerance.

### Disturbance of *RPS1* Expression Destabilizes Thylakoid Membranes

**Figure 6:**
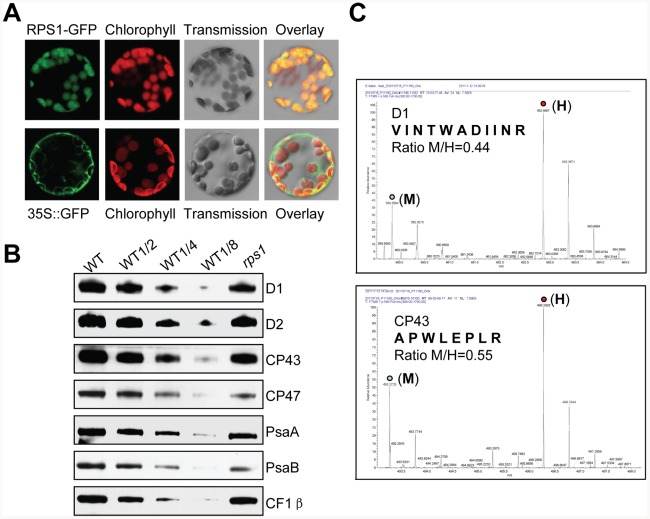
RPS1 functions in biosynthesis of chloroplast proteins. (A) RPS1-GFP signals in mesophyll cell protoplasts. (B) Western blot analysis of thylakoid membrane proteins extracted from WT and *rps1* leaves. Equal protein loading was determined by contents (2 µg) of chlorophyll in thylakoid membrane extracts according to [106]. (C) The representative mass spectra for identification of D1 and CP43 proteins in samples extracted from wild type and *rps1* mutant leaves pulse-labeled with “heavy” (H) and “medium heavy” (M) stable isotope amino acids, respectively. The ratio of peak intensities of H versus M peptides reflects difference between wild type and *rps1* mutant in translation of the corresponding proteins since the newly synthesized proteins incorporate either the H or M amino acids.

It is well known that heat stress leads to a loss of thylakoid membrane integrity, especially destacking of thylakoid membranes [2], [12]–[14], suggesting that the maintenance of thylakoid stability in response to heat stress is a key sign of heat tolerance in plants. To confirm that RPS1 is the chloroplast orthologue of CS1 in spinach [61], and CreS1 in *C. reinhardtii* [65], we determined its localization in mesophyll cell protoplasts and guard cells prepared from the transgenic plant leaves harboring *35S:RPS1-GFP* constructs and its role in synthesis of thylakoid membrane proteins encoded by chloroplast genes. As predicted, RPS1 is targeted to chloroplasts (Figure 6A and Figure S11) and the downregulation of *RPS1* in the *rps1* mutant caused a substantial reduction (50–60%) in the protein levels of thylakoid membrane proteins, represented by D1, D2, CP43, CP47, PsaA, PsaB and β-subunits of ATPase (Figure 6B). To further determine the function of RPS1 in plastid protein translation, we examined the differences in translation efficiency of thylakoid membrane proteins encoded by chloroplast DNA between wild type and the *rps1* mutant by employing the pulsed stable isotope labeling assay with amino acids [70]. As shown in Figure 6C, the translation efficiency of D1 and CP43 proteins in chloroplast was reduced by 56% and 45% respectively in *rps1* mutant leaves incorporated with medium heavy isotope-labeled amino acids (M), compared with that in wild type leaves incorporated with heavy isotope-labeled amino acids (H). The ratio of peak intensities of M versus H peptides reflects difference in translation of the corresponding proteins D1 and CP43 between wild type and the *rps1* mutant. These results demonstrate that RPS1 plays a critical role in biosynthesis of thylakoid membrane proteins encoded by chloroplast genes.

**Figure 7:**
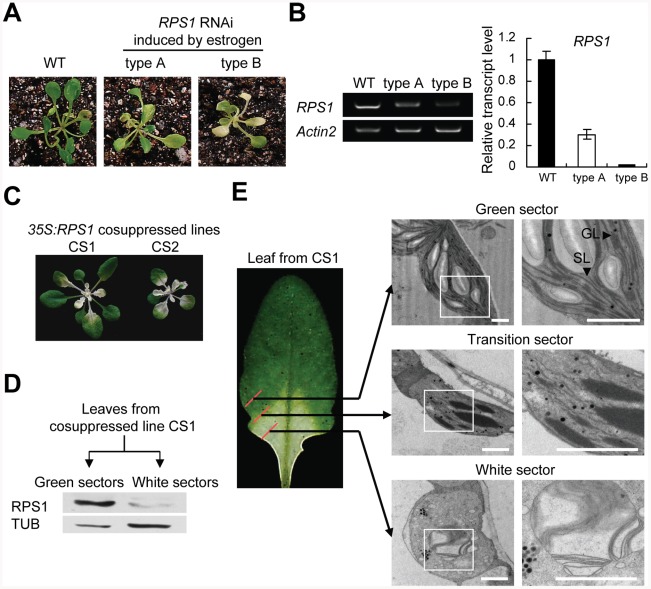
Disturbance of *RPS1* expression destabilizes thylakoid membranes. (A) Estrogen-induced variegated phenotypes of transgenic seedlings harboring estrogen-inducible *RPS1-*RNAi constructs. (B) *RPS1* mRNA levels in leaves of the transgenic plants as described in (C) were examined by RT-PCR (left) and qRT-PCR (right). For qRT-PCR analysis, *Actin2* was used as the internal standard. Error bars indicate standard deviations of three technical replicates, and the results were consistent in three biological replicates. (C) *35S:RPS1* cosuppressed lines showing a variegated phenotype. (D) RPS1 protein levels in green and white sectors excised from leaves of cosuppressed lines were examined by western blot analysis with an RPS1 polyclonal antibody. Equal protein loading was confirmed with antiserum against α-Tublin. (E) Cross-sectional analysis of thylakoid membranes in chloroplasts from the green sector, transition section, and white sector of a leaf excised from *35S:RPS1* cosuppressed line CS1 by transmission electron microscopy (TEM). Bars = 1 µm.

Having established that RPS1 functions in synthesis of thylakoid membrane proteins, we next investigated whether the downregulation of *RPS1* altered the stability of thylakoid membranes. We generated estrogen-inducible *RPS1-*RNAi and *35S:RPS1* cosuppressed transgenic lines (CS1 and CS2) and in both cases the transgenic plants exhibited a variegated phenotype (Figure 7A and 7C). We attempted to examine the phenotypes of *RPS1* overexpression transgenic lines and were not able to obtain such overexpression lines mainly owing to co-suppression caused by overexpressing *RPS1* under the control of constitutive CaMV35S promoter. Estrogen treatment-induced downregulation of *RPS1* expression was validated in estrogen-inducible *RPS1-*RNAi lines using RT-PCR and qRT-PCR (Figure 7B). Furthermore, western blots with polyclonal antisera against RPS1 confirmed the highly reduced levels of *RPS1* in the white sectors but the relatively higher levels in the green sectors of the representative variegated CS1 leaves (Figure 7D). Detailed examinations of a representative variegated leaf detached from a mature CS1 plant using transmission electron microscopy (TEM) revealed that chloroplast structures in the green sector had normally developed granal stacks, but granum-stroma thylakoid membranes were severely disrupted in the transition sector with broken stromal membranes and large, thick granal stacks; the configurations of thylakoid systems nearly disappeared in the white sector, with the thylakoids decomposed into vesicles (Figure 7E). These results further support the conclusion that RPS1 plays a critical role in synthesis of thylakoid membrane proteins that are required for maintaining the stability and integrity of thylakoid membranes in a RPS1 expression level-dependent manner.

### Overexpression of *HsfA2* in *rps1* Mutant Restores Heat-Susceptible Thylakoid Stability to Wild-Type Levels

**Figure 8:**
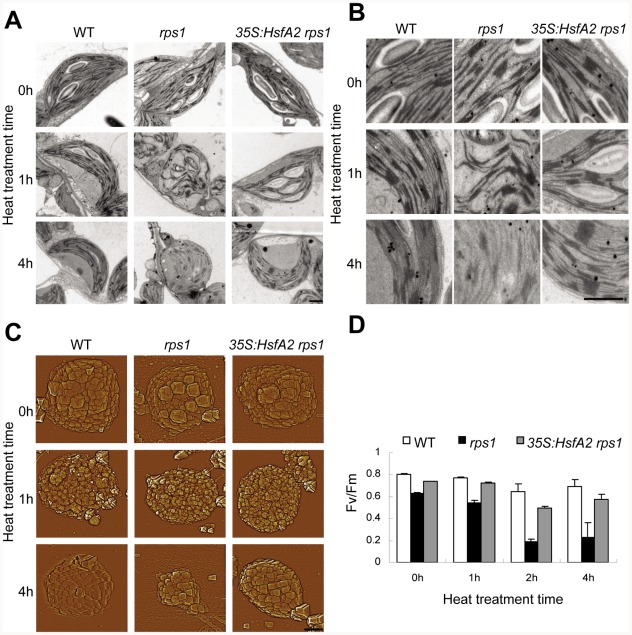
Overexpression of *HsfA2* recovers *rps1* in thylakoid stability. (A) Low magnification overview of the chloroplast ultrastructure from detached, fully expanded leaves challenged with heat treatment (38°C) for the indicated time in dark by TEM analysis. The leaves were excised from 21-d-old plants of WT, *rps1* and *rps1* with a *35S:HsfA2* cDNA transgene. Bars = 1 µm. (B) Close-up images showing details of granum and stroma thylakoids in chloroplasts as indicated in (A). (C) Atomic force microscopy (AFM) images showing granum structure of de-enveloped chloroplasts isolated from detached leaves heated for the indicated time as shown in (B). Bars = 2 µm. (D) Photochemical efficiency of PSII (Fv/Fm) of detached fifth leaves challenged with heat treatment (38°C) for the indicated time in dark. The fifth leaves were excised from WT, *rps1* and *rps1* with a *35S:HsfA2* cDNA transgene plants (21-d-old). Error bars indicate standard deviations (n = 6).

As the most sensitive component to the inhibiting action of heat stress in chloroplasts, thylakoid membrane system is vulnerable to be destabilized under heat stress conditions [2]. We next addressed whether the restoration of heat tolerance in the *rps1* mutant by *HsfA2* overexpression would be correlated with the improvement in thylakoid membrane stability under heat stress. To this end, we conducted TEM examinations. The thylakoid membranes in the chloroplasts of the *rps1* mutant appeared distorted (38°C for 1 h) and began to decompose 4 h after heat stress, whereas the wild type thylakoid systems, including grana and stroma membranes, retained their initial configurations (Figure 8A and 8B). More importantly, overexpressing *HsfA2* restored thylakoid stability in the *rps1* mutant to wild type levels (Figure 8A and 8B). To further corroborate TEM ultrastructural findings, we studied the surface topography of thylakoids in de-enveloped chloroplasts by atomic force microscopy (AFM). AFM images also revealed that heat stress led to a considerable loss of granum structures in de-enveloped chloroplasts from the *rps1* mutant, observed as a substantial reduction in the size of de-enveloped chloroplasts, but little reduction was observed for wild type and the *rps1* mutant with overexpressed *HsfA2* (Figure 8C). These observations further confirmed that the *rps1* mutant thylakoid membranes are susceptible to heat stress, which is mainly caused by the inhibition of the heat-responsive activation of *HsfA2*-dependent heat tolerance pathway. Furthermore, the heat-induced dramatic decrease in Fv/Fm values in the *rps1* mutant was reverted by overexpressing *HsfA2*, reflecting a substantial improvement in thylakoid stability and indicating that *HsfA2*-dependent restoration of thylakoid stability in the mutant is physiologically relevant (Figure 8D). Therefore, these findings have established a previously unrecognized genetic connection between the *RPS1* expression in chloroplast and the activation of *HsfA2*-dependent heat-responsive gene expression in nucleus, which is required for heat tolerance in higher plants.

## Discussion

In recent years, proteomics has provided a powerful approach to discovering the genes and pathways that are crucial for heat stress responsiveness in a variety of plant species [71]. In contrast to many studies on analysis of Arabidopsis transcriptome in response to heat stress [18]–[23], a few reports have focused on proteome analysis of heat stress-responsive proteins in *Arabidopsis* [72], [73]. In this study, we have demonstrated that RPS1 is a heat-responsive protein based on two lines of evidence from proteomic screen and western blot analysis with a RPS1 polyclonal antibody. Although numerous studies on the proteomic response to heat shock were reported on a variety of plant species such as rice, wheat, barley and *Arabidopsis* [71], the physiologically relevant roles of the proteins identified in these studies have hardly been examined by analyzing heat stress-related phenotypes of corresponding mutant plants. This difficult situation is probably due to the limited mutant resources and the functional redundancy for most of the heat-responsive genes such as *HSPs* [16], [17]. Fortunately, we identified a homozygous knockdown mutant of *rps1*, which enabled us to do reverse genetic analysis to define the entirely novel roles of RPS1 in cellular heat stress response. We have answered the question of whether downregulation of *RPS1* expression alters plant responses to heat stress by studying the knockdown mutant of *rps1* and *RPS1*-RNAi transgenic plants; both the mutant and RNAi plants displayed sensitivity to heat stress. These results provide strong genetic evidence to support that RPS1 is required for heat tolerance.

It is generally accepted that the chloroplasts in modern plants and algae are the descendants of the ancient photosynthetic bacteria. The modern chloroplast maintains a circular genome and transcription and translation machinery similar to that of its evolutionary precursor [74], [75]. In *E. coli*, ribosomal protein S1 contains six S1 domains that are essential for RNA binding and is an essential protein required for the translation of most transcripts [57], [59], [60]. Based on the molecular diversity analysis, RPS1s in prokaryotes have been classified into four types depending on their functional reliability of translation initiation [58]. According to the complete proteome of chloroplast ribosomes from higher plants [76], [77], a majority of the protein components of chloroplast ribosomes have clear homologs in bacterial 70S ribosomes. In this study, we have cloned Arabidopsis RPS1 that contains three S1 domains and shares high sequence similarity with CS1 in spinach [78], but a much less similarity with CreS1 in *C. reinhardtii* [65] and RPS1 in cyanobacteria [63] (Figures S3 and S12). Up to the present, the studies on CS1 and CreS1, the orthologues of RPS1 in spinach and *C. reinhardtii*, have been limited to their expression and RNA binding properties [62], [65], [78]. Considered that RPS1, a chloroplast-localized protein, has previously not been associated with heat stress responses, we have highlighted RPS1 as a possible candidate protein for further functional analysis of its role in heat tolerance.

With reference to the roles of RPS1 orthologues in prokaryotic and eukaryotic organisms, we had expected RPS1 to be essential in synthesis of photosynthetic proteins encoded by chloroplast genome in *Arabidopsis*. As expected, we have provided strong evidence of a functional role for RPS1 in synthesis of thylakoid membrane proteins that are needed for maintaining the stability of thylakoid membrane system in chloroplasts of plants under normal growth conditions. Importantly, TEM examination of chloroplast structures in the green, transition and white sectors sampled from a representative variegated leaf detached from cosuppressed transgenic line CS1 reveals that the protein level of RPS1 positively correlates with the de-organization degree of thylakoid membrane systems in chloroplasts (Figure 7). Knockdown of *RPS1* impairs the integrity of chloroplast as evidenced by alterations in the microscopic ultrastructures of chloroplast in the *rps1* mutant with the reduced *Fv/Fm* value under control condition (Figure 8). Throughout the molecular, biochemical and microscopic analysis, we conclude that RPS1 plays a critical role in maintaining the chloroplast integrity. Such a conclusion was drawn on the basis of reverse genetics analysis using the knockdown mutant of *rps1*, *RPS1*-RNAi transgenic plants and *35S:RPS1* cosuppression lines.

In addition to identification of RPS1 as a heat-responsive protein, it truly surprised us to find that knockdown of *RPS1* expression almost eliminates the transcriptional activation of *HsfA2* and its target genes in the *rps1* mutant in response to heat stress. It is known that translation impairment in plastids leads to downregulation of nuclear photosynthetic genes in higher plants [79]–[81]. Given that RPS1 functions in the translation initiation of chloroplast proteins and determines the integrity of chloroplast, we have proposed that the maintenance of chloroplast integrity is required for initiating the many molecular processes that signal the transcriptional activation of *HsfA2* and its target genes required for establishing cellular heat tolerance. Accordingly, the exchange of signals is required for coordination between the activities of organelles and the nucleus. However, the mechanisms that generate the retrograde signal(s) to activate the expression of heat-responsive genes in plants remain to be characterized. Numerous studies suggest the existence of plastid signals passing from the chloroplast to the nucleus [80]. Mg-protoporphyrin IX was identified as a negative signal generated from defective plastids to repress the expression of photosynthetic genes in the nucleus [82], [83]. In *C. reinhardtii*, it was reported that Mg-protoporphyrin IX could induce the expression of nuclear chaperone genes *HSP70A* and *HSP70B* [84]. However, recent reports have shown that the repression of photosynthetic gene expression caused by defective plastids has no correlation with the steady-state levels of Mg-protoporphyrin IX [85], [86]. Instead of the tetrapyrrole pathway, it is proposed that plastid signals could derive from various sources, including protein synthesis, reactive oxygen species, or the redox state of the organelle, but the identity of the putative organellar signaling molecules remains elusive [87].

Since the components of the photosynthetic apparatus housed in the chloroplasts, including the oxygen evolving complex along with the associated cofactors in PSII, carbon fixation by Rubisco and the ATP generating system, are the primary susceptible targets of thermal damage in plants, the chloroplasts were proposed as sensors to changes in the growth environment, especially to a shift up in temperature [2], [88]. In this study, we have demonstrated that the retrograde activation of *HsfA2* expression is required for maintaining the integrity of chloroplasts indicated as the stability of thylakoid membrane systems under heat stress. We have drawn this conclusion based on several lines of evidence. Firstly, knockdown of *RPS1* inhibits the heat-responsive activation of *HsfA2* and its target genes and leads to a heat-sensitive phenotype of the *rps1* mutant plants. Secondly, the overexpression of *HsfA2* is sufficient to reestablish the lost heat tolerance in the *rps1* mutant plants (Figure 4). Thirdly, the overexpression of *HsfA2* and its target genes in the *rps1* mutant dramatically improves the stability of thylakoid membranes under heat stress, which contributes to the restoration of heat tolerance in the *rps1* mutant plants (Figure 5 and Figure 8). Importantly, it should be noted that the transcriptional and protein levels of HSP21, encoded by the HsfA2 target gene *Hsp25.3-P*, are enhanced dramatically in *35S:HsfA2 rps1* plants in comparison with wild type and the *rps1* mutant plants (Figure 5). These data may explain why the heat-sensitive integrity of the *rps1* mutant chloroplasts is reverted by overexpressing *HsfA2* since previous studies indicate that HSP21 is targeted to chloroplast and functions in protecting chloroplasts from heat or oxidative stresses in higher plants [34], [35].

In general, the central message of our study is that the translation defects caused by downregulation of RPS1 in chloroplast negatively modulate nuclear heat-responsive gene expression under heat stress, leading to a loss of heat tolerance in the mutant plants, which reveals the existence of a retrograde activation pathway for cellular heat response in *Arabidopsis*. Consistent with the model we propose for the chloroplast regulation of the cellular heat stress responses, we found that knockdown of *RPS17*, an essential subunit of chloroplast ribosome since the knock-out mutant of its maize orthologue *hcf60* is seedling-lethal [89], also led to a significant reduction in the heat-responsive expression of *HsfA2* and a heat-sensitive phenotype in the *rps17* mutant plants (Figure S13). In addition, the treatment with lincomycin, an inhibitor of the chloroplast protein synthesis, severely inhibited the expression of *HsfA2* in response to heat stress (Figure S14). These additional data have further confirmed that cellular heat responses are modulated by a retrograde activation pathway in *Arabidopsis*.

Our findings have revealed that RPS1 is a key genetic connection between chloroplast translation capacity and the heat-responsive transcriptional activation of *HsfA2*, which helps in better understanding of the complex regulatory network of *HsfA2* with a new angle. As a key component of the HSF signaling network involved in cellular heat stress responses, the regulatory mechanisms of *HsfA2* have been intensely studied. In *Arabidopsis*, recent studies indicate that HsfA1 transcription factors, including HsfA1a, HsfA1b, HsfA1d, HsfA1e, function as the main regulators in heat-responsive gene expression such as *HsfA2* [52]–[54]. Interestingly, we found no significant difference in the expression levels of *HsfA1s* between wild type and the *rps1* mutant in response to heat stress (Figure S10).

According to existing literatures and our data presented in this study, we favor the model that the capacity of protein translation in chloroplasts plays a critical role in generating the retrograde signal (s) to activate the heat-responsive expressions of *HsfA2* and its target genes. We have demonstrated that RPS1 determines the stability of thylakoid membranes (Figure 7) by modulating the translational efficiency of thylakoid proteins encoded by the chloroplast genes (Figure 6). It is well known that the chloroplasts are major sites of the production of reactive oxygen species (ROS) [90]. ROS are proposed to diffuse away from their sites of production and consequently elicit a different set of signaling events under a wide range of biotic and abiotic stress conditions [91], [92]. We speculate that the alterations of thylakoid membranes caused by the downregulation of RPS1 may affect the generation of ROS such as H_2_O_2_ under heat stress by inhibiting thylakoid membrane-associated physiological processes, which could repress the activation of ROS-mediated retrograde signal transduction. Indeed, H_2_O_2_ is thought to be a signaling molecule to activate the core transcription regulators in response to heat stress. Interestingly, the exogenous application of H_2_O_2_ induces the expression of *HSP* genes in plant cells [93], [94]. It has been suggested that the HsfA4a acts as a H_2_O_2_ sensor in controlling the homeostasis of reactive oxygen species in higher plants [95]. In Class A *HSFs*, *HsfA2* is shown to have the highest level of expression in response to heat stress and the treatments with H_2_O_2_ and ozone [49]. Studies have pointed to a critical role of the mitogen-activated protein kinase (MAPK) in H_2_O_2_-mediated expression of *HSFs*, including *HsfA2* under heat stress [16], [96]–[99]. In this view, it has been suggested that H_2_O_2_ diffuses freely across the chloroplast envelope to activate a cytosolic MAPK cascade [100]. It is assumed that H_2_O_2_ may regulate the activity of HsfA1s through the mitogen-activated protein kinase [97], [98] or Calmodulin (CaM)-binding protein kinase 3 (CBK3) [101] pathways. Consequently, the activated HsfA1s regulate the heat-responsive expression of *HsfA2* and its target genes, which is required for heat tolerance. Although H_2_O_2_ is proposed as a possible retrograde signal molecule, the difficulty with this model lies in that how H_2_O_2_ could specifically communicate information on the state of chloroplasts to the nucleus because H_2_O_2_ is produced at different sites in the cell and in response to various different stresses and stimuli in higher plants [87]. Therefore, these proposed pathways remain to be further explored. The future studies should turn towards the identification of interconnecting components between the capacity of plastid protein translation and the nuclear heat-responsive gene expression of *HsfA2* and its target genes.

In summary, by integrating a variety of approaches, including proteomics, reverse genetics and microscopic analysis (TEM and AFM), we have identified RPS1 as a previously unrecognized determinant regulator involving in plastid protein translation control and retrograde activation of heat-responsive genes. Our findings demonstrate the existence of a retrograde pathway in the regulation of the cellular heat stress responses. In this view, the maintenance of chloroplast integrity under heat stress is a highly coordinated process in which RPS1 is a previously unrecognized regulator that optimizes the adaptive value of the cellular heat stress response in correspondence to capacity of plastid protein translation.

## Materials and Methods

### Plant Material and Growth Conditions

*Arabidopsis thaliana* plants used in all experiments were of the ecotype Columbia (Col-0). Growth chambers were used for controlled temperature experiments. All seeds were surface-sterilized, plated on half-strength MS medium, and stratified at 4°C for 3 days or grown in soil-culture without sterilization. Plants were grown under long-day conditions, 16 h of white light (80 µmol m^−2^ s^−1^) and 8 h of dark, with 60% relative air humidity at 21°C. The *rps1* mutant was isolated from the T-DNA insertion line (CS874869) obtained from the Arabidopsis Biological Resource Center (Ohio State University, USA). The *rps1* mutant was backcrossed to wild type twice for removing background mutations and the heat sensitive phenotype of F_2_ backcrossed lines co-segregated with T-DNA insertion as a single recessive trait.

### Plasmid Constructions and Plant Transformations

For subcellular localization analysis, a cDNA clone containing the full-length *RPS1* open reading frame was amplified by PCR with RGFP+ and RGFP- primers and inserted into *Xho*I and *Spe*I cloning sites of the 35S CaMV expression cassette of the p35S-GFP-JFH1 vector [102], yielding a C-terminal GFP fusion construct. For the genomic complementation assay, a genomic fragment of *RPS1* (4,597 bp in size), starting at 2,139 bp upstream of the ATG codon and ending at 496 bp after the stop codon, was amplified from genomic DNA by PCR with RComp+ and RComp− primers and cloned into *Kpn*I and *Xba*I sites of the pCAMBIA1300 binary vector. For the expression pattern analysis, a promoter fragment extending 2139 bp upstream to 201 bp downstream of the translation initiation ATG codon of *RPS1* was amplified using the primers RGUS+ and RGUS− and the resulting fragment was cloned into *HindIII* and *BamHI* sites of the binary vector pBI101.1. To generate the constitutive *RPS1*-dsRNAi construct and estrogen-inductive *RPS1*-dsRNAi construct, a 120 nucleotide intron of the *AtRTM1* gene [103] was subcloned into *Xba*I and *Not*I sites of the pBluescript SK+ (Stratagene, La Jolla, CA) vector to create pBS-RTM. A 485-bp sense fragment, starting at 25 bp upstream and ending at 457 bp downstream of the ATG codon of *RPS1*, was amplified with FRNAi+ and FRNAi− primers, and the antisense fragment was amplified with RRNAi+ and RRNAi− primers. The amplified sense and antisense fragments were subcloned into pBS-RTM to yield the pBS-RPS1-RTM-1SPR vector. To generate the constitutive *RPS1*-dsRNAi construct, the RPS1-RTM-1SPR fragment was released from the pBS-RPS1-RTM-1SPR vector by digestion with *Sma*I and *Sac*I and cloned into pCAMBIA1300s to generate pCAMBIA1300s-dsRPS1. For the estrogen-inductive *RPS1*-dsRNAi construct, the RPS1-RTM-1SPR fragment was obtained by digesting the pBS-RPS1-RTM-1SPR vector with *Xho*I and cloned into pER8 to generate pER8-dsRPS1. The pER8 vector [104] was kindly provided by N.-H. Chua (Rockefeller University, New York, USA). For overexpressing *HsfA2* in the *rps1* mutant, a cDNA clone containing the full-length *HsfA2* open reading frame was amplified by PCR with 121A2-F and 121A2-R primers and inserted into *Xba*I and *Sac*I cloning sites in the 35S CaMV expression cassette of pBI121. For overexpression of *RPS1* in transgenic plants, a cDNA clone containing the full-length *RPS1* open reading frame was amplified by PCR with ROX+ and ROX− primers, digested with *BamH*I and *Kpn*I and inserted into *Bgl*II and *Kpn*I cloning sites in the 35S CaMV expression cassette of pMON530. The co-suppression line CS1 and CS2 were isolated from the resulting overexpression lines described above. The primer sequences for generating the indicated constructs are listed in Table S1.

Binary vectors harboring the desired constructs were transferred into *Agrobacterium tumefaciens* strain GV3101. Transgenic plants were generated by a floral dip method and screened on solid plates containing 50 mg/L kanamycin or 25 mg/L hygromycin.

### Heat Stress Treatments

Heat tolerance assays of seedlings, mature plants and detached leaves were performed as described previously [30], [105] with modifications. All heat treatments were performed in dark. For the acquired heat tolerance test of seedlings, 2.5-d-old seedlings, grown on 1/2 MS medium, were initially acclimated to heat at 38°C for 1 h, returned to 22°C for 2 h, and then challenged at 45°C for 3 h. Challenged Seedlings recovered in a growth chamber at 22°C for 7 d under 16/8 h light-dark cycles. To evaluate the effect of continuous moderate heat stress on mature plants, 21-d-old plants grown in peat soil pots were heated in the incubator at 38°C for 9 h and the challenged plants were allowed to recover at 22°C for 3 d under continuous light conditions. For the heat tolerance assay of detached leaves, fully extended leaves detached from 21-d-old plants were placed on plastic square Petri dishes with three-layer Whatman filter paper at the bottom immersed in 20 ml of deionized water, incubated at 38°C for 6 h, and the challenged leaves were allowed to recover at 22°C for 3 d under continuous light conditions. For all heat treatments, plants were photographed following the recovery processes.

### Quantitative Real-Time RT–PCR

Total RNA was isolated from leaf samples frozen in liquid nitrogen using the TRIzol reagent (Takara) according to manufacturer's protocol. For quantitative real-time RT-PCR analysis, DNA contaminated in total RNA samples was digested with RNase-free DNase (Takara). Complementary DNA was produced using 1 µg total RNA and an oligo (dT) 18 primer. Quantitative real-time PCR was performed with SYBR Premix Ex TaqII (Takara) using a MyiQ5 single color Real-Time PCR Detection System (Bio-Rad). The comparative threshold cycle (Ct) method was used for determining relative transcript levels (iQ5 admin, Bio-Rad) using *ACTIN2* as an internal control. Three biological and three technical repeats were performed in the experiments. Primer names and sequences are listed in Table S2.

### Thylakoid Membrane Preparation and Western Blot Analysis

Thylakoid membranes were prepared as described previously [106]. Immunodetection of thylakoid membrane proteins was performed using the indicated primary antibodies against thylakoid membrane proteins (Agrisera) and Alkaline-phosphatase-conjugated goat anti-rabbit IgG (Chemicon) as a secondary antibody and reaction was revealed using an ECL kit (Amersham).

### Transmission Electron Microscopy

Samples were fixed with 2.5% (v/v) glutaraldehyde and 2% (v/v) paraformaldehyde for approximately 4 h at 4°C. Thin sections were examined by a transmission electron microscope (H7650, Hitachi) using a voltage of 120 kV.

### Protein Extraction and 2-DE Analysis

Proteins were extracted from detached, fully-extended wild type leaves treated with control or heat treatment (38°C, 2 h) in dark. Proteins were extracted and 2-DE analysis was performed as described previously [107]. Second dimension SDS-PAGE was performed using a 12% acrylamide gel in a PROTEAN II xi Cell system (Bio-Rad). Silver-stained gels were digitized by an FLA-7000 imaging analyzer (Fujifilm) and analyzed using Multi Gauge software (Fujifilm).

### MALDI-TOF/TOF MS Analysis

Mass spectra were measured using a 4700 proteomic analyzer MALDI-TOF/TOF tandem system (Applied Biosystems, Framingham, MA, USA). Gel pieces were detained with a solution of 15 mM potassium ferricyanide and 50 mM sodium thiosulfate (1∶1) for 20 min at room temperature, washed twice with deionized water, and shrunk by dehydration in ACN. The samples were swollen in a digestion buffer containing 20 mM ammonium bicarbonate and 12.5 ng/L trypsin at 4°C for 30 min and then digested more than 12 h at 37°C. Peptides in the samples were extracted twice using 0.1% TFA in 50% ACN. The extracts were dried under the protection of N_2_. For MALDI-TOF-MS, the peptides were eluted onto the target with 0.7 µl matrix solution (α-cyano-4-hydroxy-cinnamic acid in 0.1%TFA, 50%ACN). Samples were allowed to dry in air before subjected to the mass spectrometer. Data from MALDI-TOF MS/MS were searched by GPS Explorer using MASCOT as a search engine.

### SDS-PAGE and Western Blots

Total protein from plants was extracted as described previously [108]. Western blotting was performed with equal amounts of protein extracts (5 µg), separated by SDS–polyacrylamide-gel electrophoresis, immunoblotted to polyvinylidene difluoride membranes (Millipore) and probed with affinity-purified RPS1 antibodies at a dilution of 1∶1,000 (v/v) in PBS buffer (pH 7.4) containing 0.05% Tween 20. Alkaline-phosphatase-conjugated goat anti-rabbit IgG (Chemicon) was used as a secondary antibody and reaction was revealed using an ECL kit (Amasham). Anti-RPS1 rabbit polyclonal antisera were generated against the peptide of RPS1 (from Ser^17^ to Leu^248^) by Abmart biomedical company (Shanghai, China). Anti-HSP21 rabbit polyclonal antisera were purchased from Agrisera (Sweden).

### In Vivo Chloroplast Protein Translation Assay

In vivo analysis of chloroplast translation was examined according to [70]. Briefly, wild type and the *rps1* mutant primary leaves detached from 15-d-old young seedlings grown in greenhouse, were vacuum-infiltrated with cycloheximide (20 mg/ml) in the incubation buffer (10 mM Tris-HCl, pH 6.8, 5 mM MgCl_2_, 20 mM KCl, and 0.1% (v/v) Tween 20) in 9 cm petri dishes with three-layer Waterman filter paper at bottom, and incubated in dark for 30 min for blocking cytosolic translation. Next, wild type and the *rps1* mutant leaves were pulse-labeled with 100 mg/L ^13^C_6_^15^N_4_ L-arginine (“heavy”, H) and 100 mg/L ^15^N_4_ L-arginine (“medium heavy”, M) in the incubation buffer, respectively, and then transferred into light condition (80 µmol•quanta m^−2^•s^−1^) at room temperature for 4 h. Thylakoid membranes were prepared from the labeled leaves as described [106]. The extracted thylakoid membranes were separated by BN-PAGE as described [109]. The thylakiod membranes samples were washed with washing buffer (50 mM BisTris-HCl, pH 7.0, 330 mM sorbitol) and then suspended in suspension buffer (25 mM BisTris-HCl, pH 7.0, 20% glycerol) at 2.0 mg chlorophyll/ml. The samples of wild type and the *rps1* mutant were mixed with equal chlorophyll quantity and the mixed sample was diluted with the equal volume of suspension buffer containing 2% (w/v) DM in a dropwise manner. After incubation at 4°C for 30 min, the insoluble material in the thylakoid samples was removed by centrifugation at 10,000 *g* for 30 min. The supernatant (5 ug chlorophyll) was mixed with one-tenth volume of 5% Serva blue G solution (100 mM BisTris-HCl, pH 7.0, 0.5 M 6-amino-n-caproic acid and 30% (w/v) glycerol) and then applied to 0.75-mm-thick 5 to 13.5% acrylamide gradient gels in a Hoefer Mighty Small vertical electrophoresis unit connected to a cooling circulator.

Gel slices in the expected molecular weight range of thylikiod complex proteins were excised, reduced, alkylated, and trypsin-digested. Extracted peptides were analyzed by LC-MS/MS on a high performance mass spectrometer (LTQ-Orbitrap XL,Thermo Finnigan, San Jose, CA). Raw data were processed using the MaxQuant 1.1.36 software package for protein identification and quantitation.

### Confocal Microscopy

GFP images were visualized by a LSM510 laser scanning confocal microscope (Zeiss, Jena, Germany) with argon laser excitation at 488 nm and a 505- to 550-nm emission filter set for GFP fluorescence.

### Preparation of De-Enveloped Chloroplasts

Envelope-free chloroplasts were prepared according to the assay described previously [110]. Briefly, fully expanded leaves detached from 3-week-old plants were challenged with heat treatment (38°C) in the indicated time, floated on icecold water for 30 min in dark and then blotted dry. The following experimental procedures were carried out in dark on ice. Next, the blotted leaves were blended in grinding buffer containing 0.4 M sorbitol, 5 mM EDTA, 5 mM EGTA, 5 mM MgCl_2_, 10 mM NaHCO_3_, 20 mM Tricine, pH 8.4, and 0.5% (w/v) fatty acid–free BSA. The resulting slurry was filtered and centrifuged for 3 min at 2600 *g* (4°C). The half of the resulting pellet (topmost) was suspended in resuspension buffer (RB; 2 mL) containing 0.3 M sorbitol, 2.5 mM EDTA, 5 mM MgCl_2_, 10 mM NaHCO_3_, 20 mM HEPES, pH 7.6, and 0.5% (w/v) fatty acid–free BSA. The suspension was centrifuged for 3 min at 200 *g*, (4°C) and the collected supernatant was then centrifuged (2600 *g* for 3 min, at 4°C) to form pellets that contained the de-enveloped chloroplasts. The resulting pellets were resuspended in RB buffer for experiments.

### Atomic Force Microscopy (AFM)

Samples were attached to glass cover slips (10 mm of diameter) coated with 0.01% (w/v) poly-L-lysine by gentle centrifugation (5 min at 1,000 *g*) and fixed for overnight at 4°C in RB containing 2% (v/v) glutaraldehyde and 3% (v/v) paraformaldehyde. Signals were recorded in contact mode with the MultiMode-SPM (Veeco Co.) equipped with a 30-µm scanner, using oxide-sharpened Si_3_N_4_ cantilevered tips (*k* = 0.12 N/m). Images were acquired with forces set minimally above lift-off values, at 1 to 2 Hz.

### Chlorophyll Fluorescence Measurements

Chlorophyll fluorescence emissions were detected with an LI-6400XT Portable Photosynthesis System (LI-COR Biosciences, Lincoln, Nebraska USA). The fifth leaves were excised from 21-d-old plants, and challenged with heat treatment (38°C) for the indicated time in dark. The maximum photochemical efficiency of PSII was determined from the ratio of variable (*Fv*) to maximum (*Fm*) fluorescence (*Fv/Fm*).

### Assays for Sensitivity to Salt and Osmotic Stresses

Measurements were performed by root-bending assay as described previously [111], [112] and by seedling-growth assay as described previously [113].

### GUS Staining

Tissues were immersed into the staining solution (50 mM Na phosphate buffer, pH 7.0, 10 mM EDTA, 0.5 mM potassium ferricyanide, 0.5 mM potassium ferrocyanide, 0.1% Triton X-100, and 2 mM 5-bromo-4-chloro-3-indolyl-ß-D-glucuronide), vacuum- infiltrated for 5 min and incubated at 37°C overnight. Stained tissues were decolorized with 70% ethanol and examined with an Olympus BX51 microscopy or Olympus SZX7 stereomicroscopy (Olympus, Japan).

### Lincomycin Treatment

Seedlings were treated with lincomycin according to [114].

### Accession Numbers

Sequence data from this article can be found in the Arabidopsis Genome Initiative or GenBank databases under the following accession numbers: *RPS1* (At5g30510), *Hsp101* (At1g74310), *Hsp70* (At3g12580), *Hsp25.3-P* (At4g27670), *Hsp18.1-CI* (At5g59720), *Hsp17.7-CII* (At5g12030), *APX2* (At3g09640), *GolS1* (At2g47180), *Actin2* (At3g18780). The National Center for Biotechnology Information accession numbers of the proteins used in Alignment analysis under the following accession numbers: CS1 in spinach (M82923) and CreS1 in *Chlamydomonas reinhardtii* (AJ585191).

## Supporting Information

Figure S1 Silver stains of 2D-PAGE electrophoresis separation of proteins in response to heat treatment. (A) Control leaves. (B) challenged leaves at 38°C for 2 h in dark. Arrows indicate the RPS1 spot. Three independent experiments were performed and showed similar patterns.
(PDF)

Figure S2 Representative tandem mass spectra for identification of RPS1 by MALDI-TOF/TOF MS analysis. Representative tandem mass spectra were showed according to precursor ions with m/z value (A) 1083.6990, and (B) 1321.7111, corresponding respectively to: (A), peptide GGLVALVEGLR, spanning residues G200 to R210 of protein RPS1 (At5g30510); (B), peptide NIQYELAWER, spanning residues N170 to R179 of protein RPS1 (At5g30510). The b ions, y ions and the resulting peptide sequences were displayed.
(PDF)

Figure S3 Alignments of derived amino acid sequences of AtRPS1, CS1 and CreS1. Amino acid sequences of AtRPS1 (At5g30510), CS1 in spinach (GenBank accession number: M82923) and CreS1 in *Chlamydomonas reinhardtii* (GenBank accession number: AJ585191) were aligned using ClustalW (http://www.ebi.ac.uk/clustalw). Alignment was shaded using BoxShade (http://www.ch.embnet.org/software/BOX_form.html). Identical amino acid residues and conservative changes were depicted in black and grey background, respectively. Three S1 domains were labeled.
(PDF)

Figure S4 Transcriptional and protein levels of *RPS1* in response to heat stress. (A) Western blot analysis showing RPS1 protein levels in wild type leaves in response to heat treatment (38°C) in dark for the indicated time with an RPS1 polyclonal antibody. Equal protein loading was confirmed with antiserum against α-Tubulin. (B) qRT-PCR analysis of mRNA levels of *RPS1* in detached, fully-extended WT leaves challenged with heat treatment (38°C) for the indicated time in dark. *Actin2* was used as the internal standard. Error bars indicate standard deviations of three technical replicates, and the results were consistent in three biological replicates.
(PDF)

Figure S5 Schematic diagram of *RPS1* gene showing the T-DNA insertion site. Open box indicates 5′ or 3′ UTR; Closed box indicates ORF. Exons (boxes) and introns (lines) were determined by a comparison of the genomic and cDNA sequences. The T-DNA insertion site and positions of the start and stop codons are indicated.
(PDF)

Figure S6 Analysis of *pRPS1:GUS* expression in transgenic plants. Transgenic Arabidopsis plants harboring *pRPS1:GUS* constructs were analyzed by GUS-staining assay. GUS-staining patterns of the representative 5-d-old (A) and 15-d-old (B) transgenic seedlings grown on half-strength MS medium.
(PDF)

Figure S7 Characterization of primary root growth of wild type and *rps1* mutant plants under salt or osmotic stress. 5-d-old wild-type and *rps1* mutant seedlings grown under normal growth conditions were transferred to MS medium containing NaCl or mannitol respectively. Phenotypes of wild type and *rps1* mutant seedlings treated with NaCl (A) or mannitol (C) were photographed and bending-growth of primary roots under salt (B) or osmotic stress (D) was measured at day 7 after transfer. Error bars represent standard deviations (n = 24 plants). Results from one of two independent experiments are shown.
(PDF)

Figure S8 Growth characterization of wild type and *rps1* mutant seedlings under salt or osmotic stress. Phenotypes of wild type and *rps1* mutant seedlings grown on MS medium with NaCl or mannitol were photographed at day 14 after germination.
(PDF)

Figure S9 Overexpression of *RPS1* improves heat tolerance in the genomic complementation lines. (A) Heat-tolerant phenotypes of the transgenic lines of *rps1* complemented with *RPS1* genomic DNA in comparison with WT seedlings challenged the heat treatment as described in Methods. (B) qRT-PCR analysis showing expression levels of *RPS1* in the genomic complementation lines as described in (A). For qRT-PCR analysis, *Actin2* was used as the internal standard. Error bars indicate standard deviations of three technical replicates, and the results were consistent in three biological replicates. (C) The ratio of peak intensities of M versus H peptides reflects difference between the genomic complementation line *Comp rps1-3* and wild type in translation of the corresponding proteins since the newly synthesized proteins incorporate either the M or H amino acids. Samples were extracted from 7-d-old seedlings of wild type and the transgenic line of *Comp rps1-3* pulse-labeled with “heavy” (H) and “medium heavy” (M) stable isotope amino acids, respectively, as described in Methods.
(PDF)

Figure S10 Heat-responsive expression analysis of HSF members in class A in wild type and *rps1* mutant plants. qRT-PCR analysis of mRNA levels of 15 class A HSF members in detached, fully-extended WT and *rps1* leaves challenged with heat treatment (38°C) for 1 h in dark. *Actin2* was used as the internal standard. Error bars indicate standard deviations of three technical replicates, and the results were consistent in three biological replicates.
(PDF)

Figure S11 Chloroplast localization patterns of RPS1-GFP in guard cells of epidermal peels. Chloroplast localization patterns were inspected with a confocal microscope. Epidermal peels were excised from the transgenic plants expressing the indicated RPS1-GFP proteins.
(PDF)

Figure S12 Phylogenetic relationships of RPS1s in *Arabidopsis thaliana* (At5g30510), *Spinacia oleracea* (accession number: M82923), *Synechocysti*s *sp. PCC 6803* (accession number: gi|1652650), *Marchantia polymorpha* (accession number: gi|786212), *Chlamydophia felis Fe/C-56* (accession number: AJ585191) and *Plasmodium chabaudi* (accession number: gi|70945988). A rooted phylogenetic tree was constructed using TreeView version 1.6.6 software with the neighbor-joining method based on ClustalW multiple alignments of the possible RPS1s. Bar = 0.1 amino acid substitutions per site.
(PDF)

Figure S13 Knockdown of *RPS17* expression in *rps17* mutant plants leads to heat susceptibility. (A) Schematic diagram of *RPS17* gene (At1g79850) showing the T-DNA insertion site. Open box indicates 5′or 3′UTR; Closed box indicates ORF. The T-DNA insertion site and positions of the start and stop codons are indicated (SALK_066943). (B) *RPS17* mRNA levels in leaves of wild type and *rps17* mutant plants were analyzed by qRT-PCR. *Actin2* was used as the internal standard. (C) Western blot analysis of thylakoid membrane proteins extracted from WT and *rps17* leaves. Equal protein loading was determined by contents (2 µg) of chlorophyll in thylakoid membrane extracts according to (Peng et al., 2006). (D) to (E) Heat-challenged phenotypes of wild type and *rps17* mutant as examined with detached leaf (D) and whole plant (E) assays performed as described in Methods. (F) qRT-PCR analysis of mRNA levels of *HsfA2* in detached, fully-extended WT and *rps1* leaves challenged with heat treatment (38°C) for the indicated time in dark. For qRT-PCR analysis, *Actin2* was used as the internal standard. Error bars indicate standard deviations of three technical replicates, and the results were consistent in three biological replicates.
(PDF)

Figure S14 Heat-responsive expression of *HsfA2* is severely inhibited in wild type seedlings by Lincomycin treatment. qRT-PCR analysis of mRNA levels of *HsfA2* in 6-d-old seedlings of WT challenged with heat treatment (38°C) for 2 h in dark under control or lincomycin treatment condition. *Actin2* was used as the internal standard. Error bars indicate standard deviations of three technical replicates, and the results were consistent in three biological replicates.
(PDF)

Table S1 Sequences of the primers for constructs.
(PDF)

Table S2 Sequences of the primers for qRT–PCR and RT–PCR.
(PDF)
