# Supplementary material for: Downregulation of Chloroplast RPS1 Negatively Modulates Nuclear Heat-Responsive Expression of HsfA2 and Its Target Genes in Arabidopsis
Source: PLoS Genet. 2012 May 3;8(5):e1002669. doi: 10.1371/journal.pgen.1002669 (PMC3342936; doi:10.1371/journal.pgen.1002669)
Supplement: Figure S1 — Silver stains of 2D-PAGE electrophoresis separation of proteins in response to heat treatment. (A) Control leaves. (B) challenged leaves at 38°C for 2 h in dark. Arrows indicate the RPS1 spot. Three independent experiments were performed and showed similar patterns. (PDF) [file pgen.1002669.s001.pdf]

Figure S1. Yu et al.

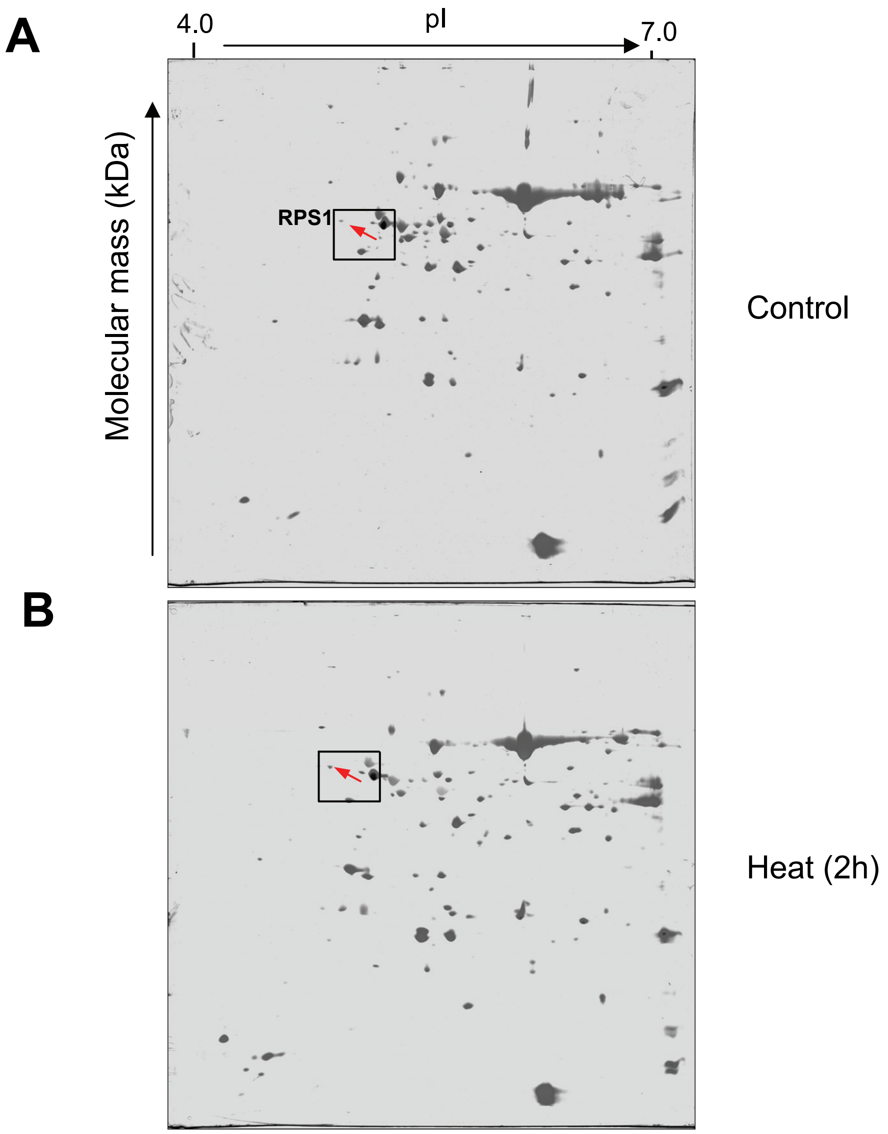

Figure S1. Silver stains of 2D-PAGE electrophoresis separation of proteins in response to heat treatment.

Proteins were extracted from control leaves (A) and the challenged leaves (B) at 38°C for 2 h in dark. The fully-expanded leaves were excised from 21-d-old wild type plants for control or heat treatment. Arrows indicate the RPS1 spot. Three independent experiments were performed and showed similar patterns.
