# Supplementary material for: Downregulation of Chloroplast RPS1 Negatively Modulates Nuclear Heat-Responsive Expression of HsfA2 and Its Target Genes in Arabidopsis
Source: PLoS Genet. 2012 May 3;8(5):e1002669. doi: 10.1371/journal.pgen.1002669 (PMC3342936; doi:10.1371/journal.pgen.1002669)
Supplement: Figure S2 — Representative tandem mass spectra for identification of RPS1 by MALDI-TOF/TOF MS analysis. Representative tandem mass spectra were showed according to precursor ions with m/z value (A) 1083.6990, and (B) 1321.7111, corresponding respectively to: (A), peptide GGLVALVEGLR, spanning residues G200 to R210 of protein RPS1 (At5g30510); (B), peptide NIQYELAWER, spanning residues N170 to R179 of protein RPS1 (At5g30510). The b ions, y ions and the resulting peptide sequences were displayed. (PDF) [file pgen.1002669.s002.pdf]

Figure S2. Yu et al.

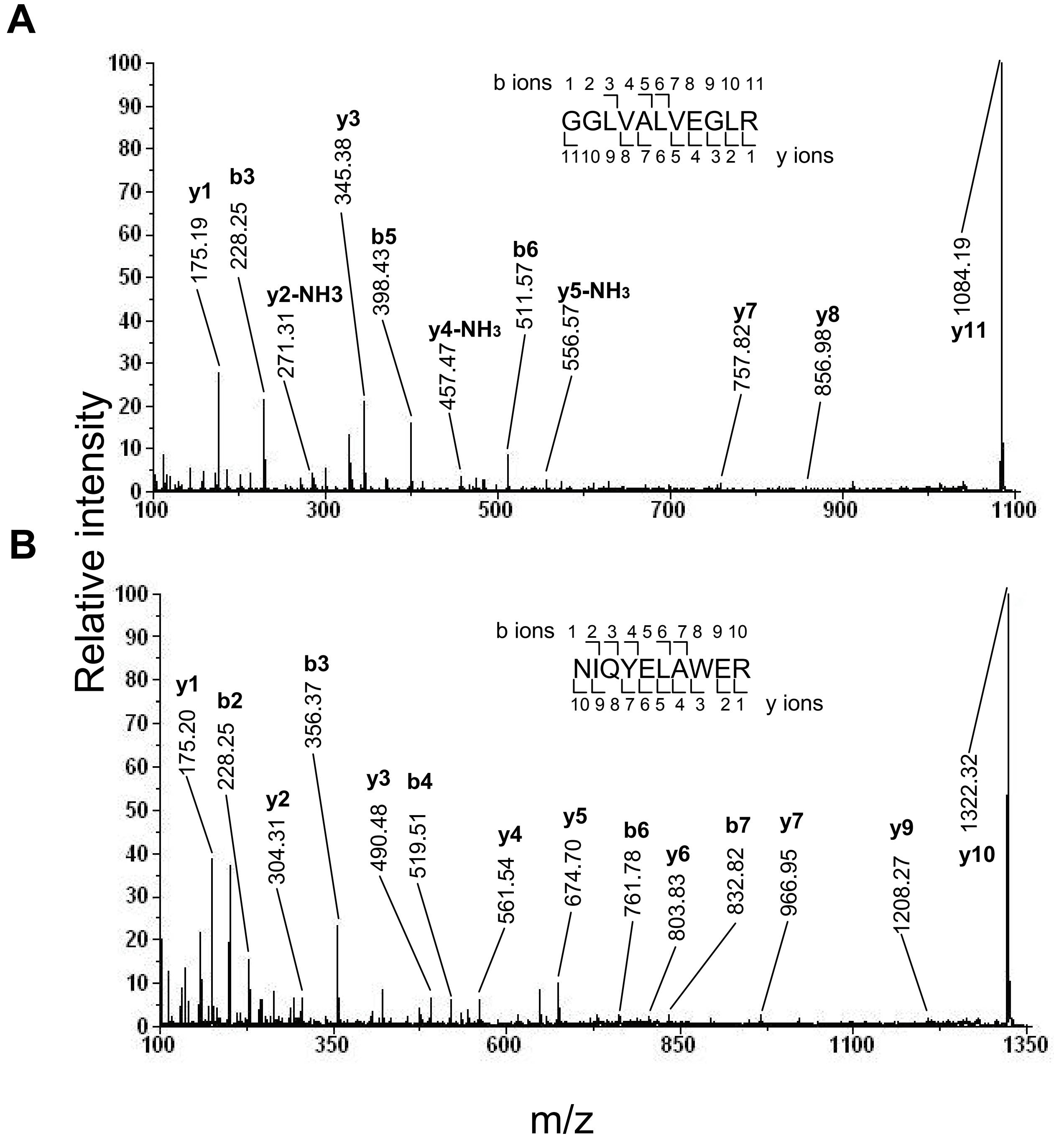

Figure S2. Representative tandem mass spectra for identification of RPS1 by MALDI-TOF/TOF MS analysis.

Representative tandem mass spectra were showed according to precursor ions with m/z value (A) 1083.6990, and (B) 1321.7111,corresponding respectively to: (A), peptide GGLVALVEGLR , spanning residues G200 to R210 of protein RPS1 (At5g30510); (B), peptide NIQYELAWER, spanning residues G170 to R179 of protein RPS1 (At5g30510). The b ions, y ions and the resulting peptide sequences were displayed.
