# Supplementary material for: Downregulation of Chloroplast RPS1 Negatively Modulates Nuclear Heat-Responsive Expression of HsfA2 and Its Target Genes in Arabidopsis
Source: PLoS Genet. 2012 May 3;8(5):e1002669. doi: 10.1371/journal.pgen.1002669 (PMC3342936; doi:10.1371/journal.pgen.1002669)
Supplement: Figure S3 — Alignments of derived amino acid sequences of AtRPS1, CS1 and CreS1. Amino acid sequences of AtRPS1 (At5g30510), CS1 in spinach (GenBank accession number: M82923) and CreS1 in Chlamydomonas reinhardtii (GenBank accession number: AJ585191) were aligned using ClustalW (http://www.ebi.ac.uk/clustalw). Alignment was shaded using BoxShade (http://www.ch.embnet.org/software/BOX_form.html). Identical amino acid residues and conservative changes were depicted in black and grey background, respectively. Three S1 domains were labeled. (PDF) [file pgen.1002669.s003.pdf]

Figure S3. Yu et al.

```
AtRPS1     1 ----MASLAQQFSG-LRCSPLSSSSRLSRRASK--------------------NFPQNKS
CS1        1 ----MASLAQQLAGGLRCPPLSNSN-LSKPFS-----------------------PKHTL
CreS1      1 MRLSARTLAQRASLPARSTGVVSRRSLVAPAPARKANLATRVAAIEEQQTSFQGDEQPVE

AtRPS1    36 ASVSPTIVAAVAMSSGQTKERLELKKMFEDAYERCRTSPMEG-----------------V
CS1       33 KPRFSPIVSAVAVSNAQTRERQKLKQLFEDAYERCRNAPMEG-----------------V
CreS1     61 AALDPASILDVDPAAVEEMDLHELDQAQEDLLKWMMLDEATQEEDLDEMVDYDDFGDEEY

                                                     S1
AtRPS1    79 AFTVDDFAAAIEQYDFNSEIGTRVKGTVFKTDANGALVDISAKSSAYLSVEQACIHRIKH
CS1       76 SFTIDDFHTALDKYDFNSEMGSRVKGTVFCTDANGALVDITAKSSAYLPLAEACIYRIKN
CreS1    121 ADMYEEVEEMLEAVDYEFKVGDKVSGTVIEVDDDGAYVEIGAKAIAFCPVTECSFARLKT

AtRPS1   139 VEEAGIVPGMVEEFVIIGENESDDSLLLSLRNIQYELAWERCRQLQAEDVIVKAKVIGAN
CS1      136 VEEAGIIPGVREEFVIIGENEADDSLILSLRQIQYELAWERCRQLQAEDVVVKGKIVGAN
CreS1    181 PLEA-LRPGMKRDFMVVEDEEEYGQVIVSLAAMEATTFWQRIRQLQEEDITVTVSVESVN

                                    S1
AtRPS1   199 KGGLVALVEGLRGFVPFSQIS---SKAAAEELLEKEIPLKFVEVDEEQTKLVLSNRKAVA
CS1      196 KGGVVALVEGLRGFVPFSQIS---SKSSAEELLEKEIPLKFVEVDEEQSRLVMSNRKAMA
CreS1    240 KGGMLVKFGIYDGFIPVSQFGPSITPDNMESLVGFELQVKFLEVDEEAERLVFSHKRASS

                                                   S1
AtRPS1   256 DSQAQLG---IGSVVLGVVQSLKPYGAFIDIGGINGLLHVSQISHDRVSDIATVLQPGDT
CS1      253 DSQAQLG---IGSVVTGTVQSLKPYGAFIDIGGINGLLHVSQISHDRVSDIATVLQPGDT
CreS1    300 TVSNDIQGFKVGDVVAGVVQSVKPYGAFIDLGGVTGLLHVSQISHDRVLVLDKILQEGDK

AtRPS1   313 LKVMILSHDRDRGRVSLSTKKLEPTPGDMIRNPKLVFEKAEEMAQTFRQRIAQAEAMARA
CS1      310 LKVMILSHDRERGRVSLSTKKLEPTPGDMIRNPKLVFEKAEEMAQTFRQRIAQAEAMARA
CreS1    360 IKVMILSQDRERGRVTLSTKKLEPTPGDMLRDPQLVYEKAEEMAEQFRKRVEQAAQSAAE

AtRPS1   373 DMLRFQPESGLTLSSDGILGPLGSELPDDGVDLTVDDIPSAVDI
CS1      370 DMLRFQPESGLTLSSDGILGPLTSDLPAEGLDLSV--VPPAVES
CreS1    420 ----PQPD---TLGAAPEGQPFVY--------------------
```

Figure S3. Alignments of derived amino acid sequences of AtRPS1, CS1 and CreS1.

Amino acid sequences of AtRPS1 (At5g30510), CS1 in spinach (accession number: M82923) and CreS1 in *Chlamydomonas reinhardtii* (accession number: AJ585191) were aligned using ClustalW (http://www.ebi.ac.uk/clustalw). Alignment was shaded using BoxShade (http://www.ch.embnet.org/software/BOX_form.html). Identical amino acid residues and conservative changes were depicted in black and grey background, respectively. Three S1 domains were labeled.
