# Supplementary material for: Downregulation of Chloroplast RPS1 Negatively Modulates Nuclear Heat-Responsive Expression of HsfA2 and Its Target Genes in Arabidopsis
Source: PLoS Genet. 2012 May 3;8(5):e1002669. doi: 10.1371/journal.pgen.1002669 (PMC3342936; doi:10.1371/journal.pgen.1002669)
Supplement: Figure S4 — Transcriptional and protein levels of RPS1 in response to heat stress. (A) Western blot analysis showing RPS1 protein levels in wild type leaves in response to heat treatment (38°C) in dark for the indicated time with an RPS1 polyclonal antibody. Equal protein loading was confirmed with antiserum against α-Tubulin. (B) qRT-PCR analysis of mRNA levels of RPS1 in detached, fully-extended WT leaves challenged with heat treatment (38°C) for the indicated time in dark. Actin2 was used as the internal standard. Error bars indicate standard deviations of three technical replicates, and the results were consistent in three biological replicates. (PDF) [file pgen.1002669.s004.pdf]

Figure S4. Yu et al.

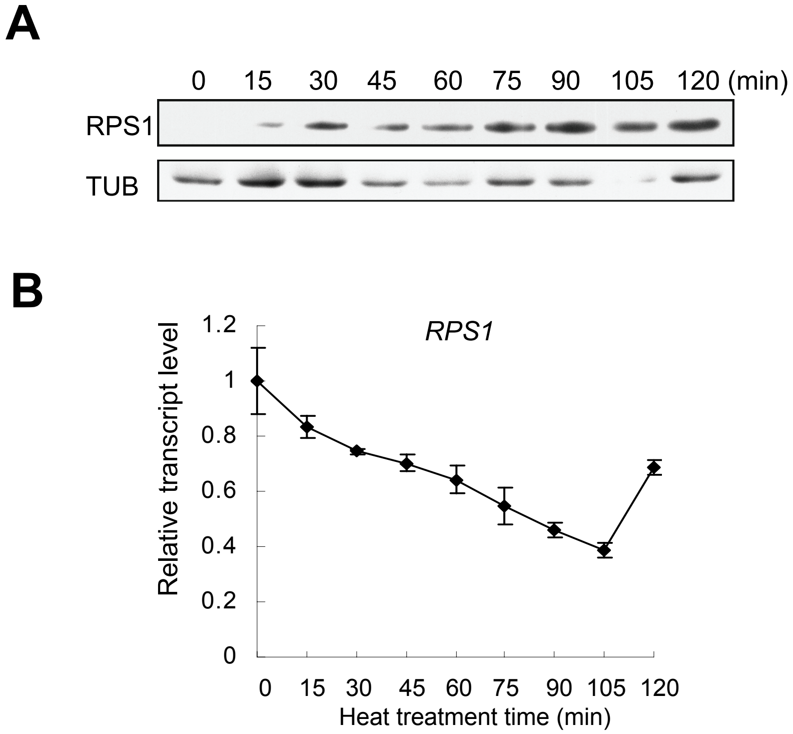

Figure S4. Transcription and protein levels of *RPS1* in response to heat stress.

(A) Western blot analysis showing RPS1 protein levels in wild type leaves in response to heat treatment (38°C) in dark for the indicated time with an RPS1 polyclonal antibody. Equal protein loading was confirmed with antiserum against α-Tubulin. (B) qRT-PCR analysis of mRNA levels of *RPS1* in detached, fully-extended WT leaves challenged with heat treatment (38°C) for the indicated time in dark. *Actin2* was used as the internal standard. Error bars indicate standard deviations of three technical replicates, and the results were consistent in three biological replicates.
