# Supplementary material for: Downregulation of Chloroplast RPS1 Negatively Modulates Nuclear Heat-Responsive Expression of HsfA2 and Its Target Genes in Arabidopsis
Source: PLoS Genet. 2012 May 3;8(5):e1002669. doi: 10.1371/journal.pgen.1002669 (PMC3342936; doi:10.1371/journal.pgen.1002669)
Supplement: Figure S5 — Schematic diagram of RPS1 gene showing the T-DNA insertion site. Open box indicates 5′ or 3′ UTR; Closed box indicates ORF. Exons (boxes) and introns (lines) were determined by a comparison of the genomic and cDNA sequences. The T-DNA insertion site and positions of the start and stop codons are indicated. (PDF) [file pgen.1002669.s005.pdf]

Figure S5. Yu et al.

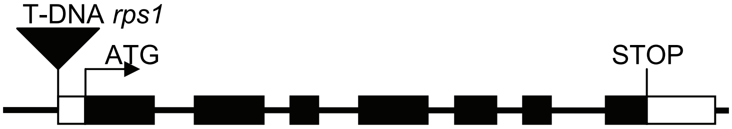

Figure S5. Schematic diagram of *RPS1* gene showing the T-DNA insertion site.

Open box indicates 5’ or 3’ UTR; Closed box indicates ORF. Exons (boxes) and introns (lines) were determined by a comparison of the genomic and cDNA sequences. The T-DNA insertion site and positions of the start and stop codons are indicated.
