# Supplementary material for: Downregulation of Chloroplast RPS1 Negatively Modulates Nuclear Heat-Responsive Expression of HsfA2 and Its Target Genes in Arabidopsis
Source: PLoS Genet. 2012 May 3;8(5):e1002669. doi: 10.1371/journal.pgen.1002669 (PMC3342936; doi:10.1371/journal.pgen.1002669)
Supplement: Figure S6 — Analysis of pRPS1:GUS expression in transgenic plants. Transgenic Arabidopsis plants harboring pRPS1:GUS constructs were analyzed by GUS-staining assay. GUS-staining patterns of the representative 5-d-old (A) and 15-d-old (B) transgenic seedlings grown on half-strength MS medium. (PDF) [file pgen.1002669.s006.pdf]

Figure S6. Yu et al.

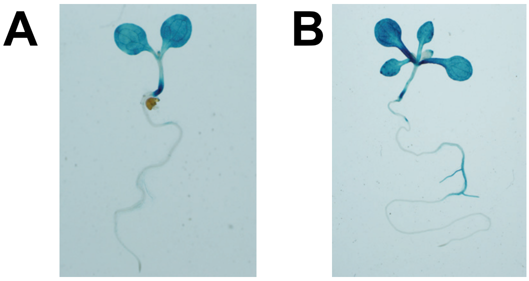

Figure S6. Analysis of *pRPS1::GUS* expression in transgenic plants.

Transgenic Arabidopsis plants harboring *pRPS1::GUS* constructs were analyzed by GUS-staining assay. GUS-staining patterns of the representative 5-d-old (A) and 15-d-old (B) transgenic seedlings grown on half-strength MS medium.
