# Supplementary material for: Downregulation of Chloroplast RPS1 Negatively Modulates Nuclear Heat-Responsive Expression of HsfA2 and Its Target Genes in Arabidopsis
Source: PLoS Genet. 2012 May 3;8(5):e1002669. doi: 10.1371/journal.pgen.1002669 (PMC3342936; doi:10.1371/journal.pgen.1002669)
Supplement: Figure S7 — Characterization of primary root growth of wild type and rps1 mutant plants under salt or osmotic stress. 5-d-old wild-type and rps1 mutant seedlings grown under normal growth conditions were transferred to MS medium containing NaCl or mannitol respectively. Phenotypes of wild type and rps1 mutant seedlings treated with NaCl (A) or mannitol (C) were photographed and bending-growth of primary roots under salt (B) or osmotic stress (D) was measured at day 7 after transfer. Error bars represent standard deviations (n = 24 plants). Results from one of two independent experiments are shown. (PDF) [file pgen.1002669.s007.pdf]

Figure S7. Yu et al.

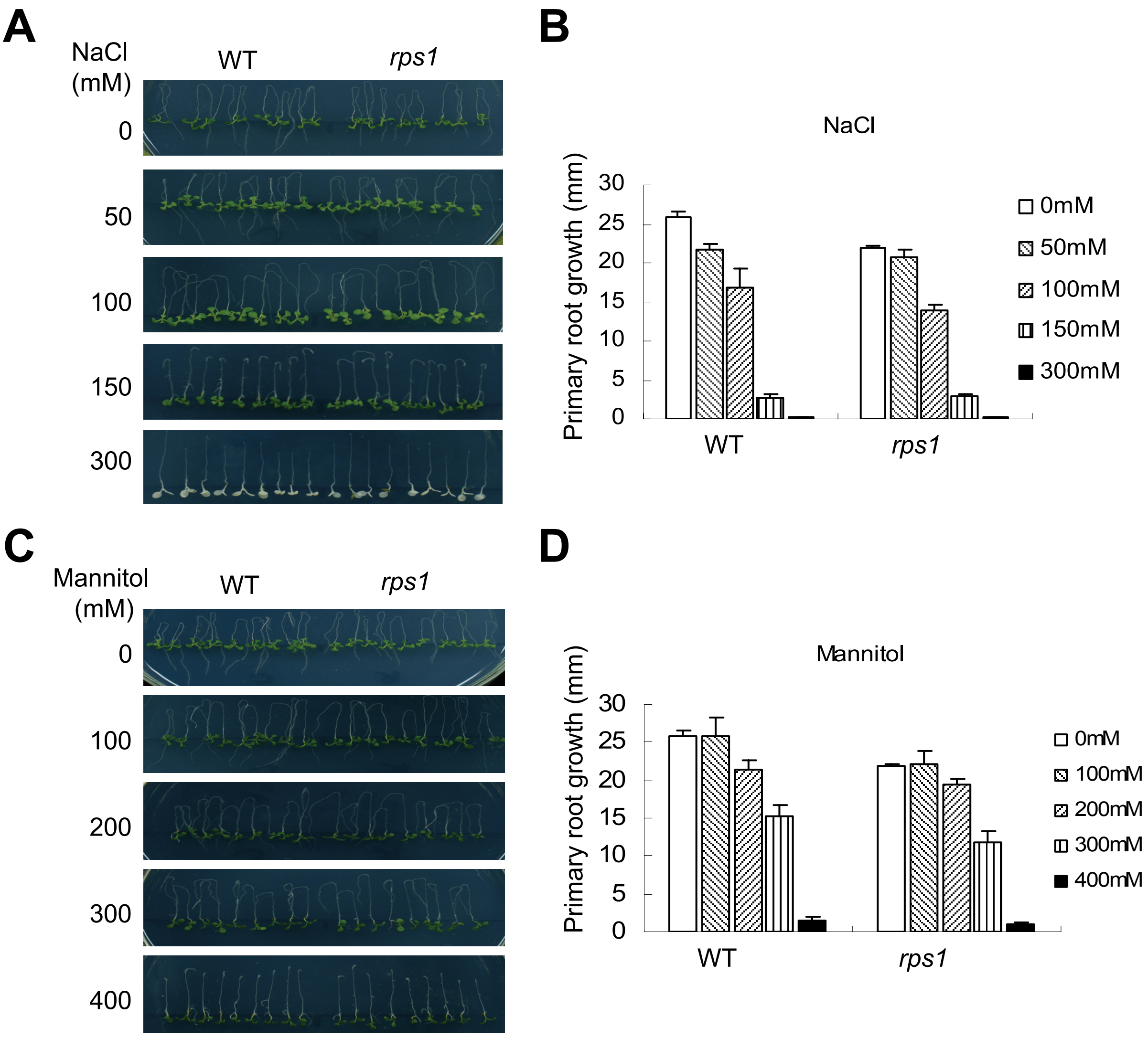

Figure S7. Characterization of primary root growth of wild type and *rps1* mutant plants under salt or osmotic stress.

5-d-old wild-type and *rps1* mutant seedlings grown under normal growth conditions were transferred to MS medium containing NaCl or mannitol respectively. Phenotypes of wild type and *rps1* mutant seedlings treated with NaCl (A) or mannitol (C) were photographed and bending-growth of primary roots under salt (B) or osmotic stress (D) was measured at day 7 after transfer. Error bars represent standard deviations (n＝24 plants). Results from one of two independent experiments are shown.
