# Supplementary material for: Downregulation of Chloroplast RPS1 Negatively Modulates Nuclear Heat-Responsive Expression of HsfA2 and Its Target Genes in Arabidopsis
Source: PLoS Genet. 2012 May 3;8(5):e1002669. doi: 10.1371/journal.pgen.1002669 (PMC3342936; doi:10.1371/journal.pgen.1002669)
Supplement: Figure S8 — Growth characterization of wild type and rps1 mutant seedlings under salt or osmotic stress. Phenotypes of wild type and rps1 mutant seedlings grown on MS medium with NaCl or mannitol were photographed at day 14 after germination. (PDF) [file pgen.1002669.s008.pdf]

Figure S8. Yu et al.

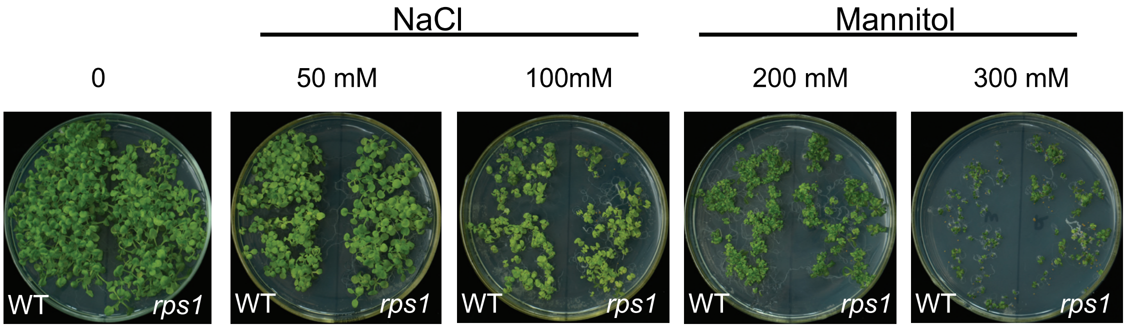

Figure S8. Growth characterization of wild type and *rps1* mutant seedlings under salt or osmotic stress.

Phenotypes of wild type and *rps1* mutant seedlings grown on MS medium with NaCl or mannitol were photographed at day 14 after germination.
