# Supplementary material for: Downregulation of Chloroplast RPS1 Negatively Modulates Nuclear Heat-Responsive Expression of HsfA2 and Its Target Genes in Arabidopsis
Source: PLoS Genet. 2012 May 3;8(5):e1002669. doi: 10.1371/journal.pgen.1002669 (PMC3342936; doi:10.1371/journal.pgen.1002669)
Supplement: Figure S9 — Overexpression of RPS1 improves heat tolerance in the genomic complementation lines. (A) Heat-tolerant phenotypes of the transgenic lines of rps1 complemented with RPS1 genomic DNA in comparison with WT seedlings challenged the heat treatment as described in Methods. (B) qRT-PCR analysis showing expression levels of RPS1 in the genomic complementation lines as described in (A). For qRT-PCR analysis, Actin2 was used as the internal standard. Error bars indicate standard deviations of three technical replicates, and the results were consistent in three biological replicates. (C) The ratio of peak intensities of M versus H peptides reflects difference between the genomic complementation line Comp rps1-3 and wild type in translation of the corresponding proteins since the newly synthesized proteins incorporate either the M or H amino acids. Samples were extracted from 7-d-old seedlings of wild type and the transgenic line of Comp rps1-3 pulse-labeled with “heavy” (H) and “medium heavy” (M) stable isotope amino acids, respectively, as described in Methods. (PDF) [file pgen.1002669.s009.pdf]

Figure S9. Yu et al.

A

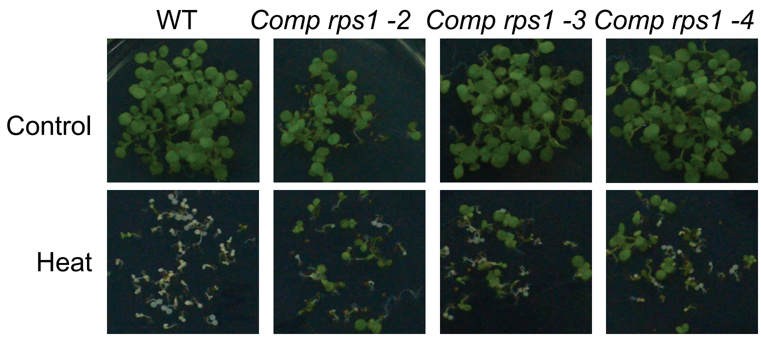

B

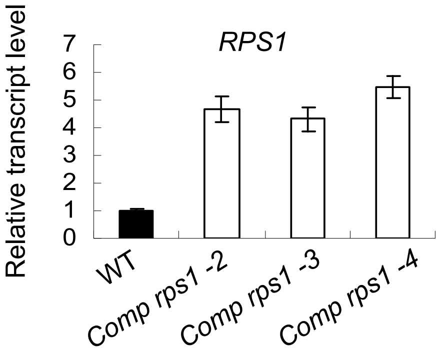

C

Protein name	Gene accession number	protein accession number	Ratio M/H Control	Ratio M/H Heat stress
**D1**	ATCG00020	gi:44889035	1.108	1.303
**D2**	ATCG00270	gi:6685787	2.155	7.259
**CP43**	ATCG00280	gi:172045852	1.131	2.690
**CP47**	ATCG00680	gi:6685792	1.475	2.305
**psaA**	ATCG00350	gi:6685788	1.172	3.439
**psaB**	ATCG00340	gi:6685789	1.310	3.515
**CF1** β	ATCG00480	gi:6686269	2.053	3.305

Figure S9. Overexpression of *RPS1* improves heat tolerance in the genomic complementation lines .

(A) Heat-tolerant phenotypes of the transgenic lines of *rps1* complemented with *RPS1* genomic DNA in comparison with WT seedlings challenged the heat treatment as described in Methods.
(B) qRT-PCR analysis showing expression levels of *RPS1* in the genomic complementation lines as described in (A).
For qRT-PCR analysis, *Actin2* was used as the internal standard. Error bars indicate standard deviations of three technical replicates, and the results were consistent in three biological replicates.
(C) The ratio of peak intensities of M versus H peptides reflects difference between the genomic complementation line *Comp rps1-3* and wild type in translation of the corresponding proteins since the newly synthesized proteins incorporate either the M or H amino acids. Samples were extracted from 7-d-old seedlings of wild type and the transgenic line of *Comp rps1-3* pulse-labeled with “heavy” (H) and “medium heavy” (M) stable isotope amino acids, respectively, as described in Methods.
