# Supplementary material for: Downregulation of Chloroplast RPS1 Negatively Modulates Nuclear Heat-Responsive Expression of HsfA2 and Its Target Genes in Arabidopsis
Source: PLoS Genet. 2012 May 3;8(5):e1002669. doi: 10.1371/journal.pgen.1002669 (PMC3342936; doi:10.1371/journal.pgen.1002669)
Supplement: Figure S10 — Heat-responsive expression analysis of HSF members in class A in wild type and rps1 mutant plants. qRT-PCR analysis of mRNA levels of 15 class A HSF members in detached, fully-extended WT and rps1 leaves challenged with heat treatment (38°C) for 1 h in dark. Actin2 was used as the internal standard. Error bars indicate standard deviations of three technical replicates, and the results were consistent in three biological replicates. (PDF) [file pgen.1002669.s010.pdf]

Figure S10. Yu et al.

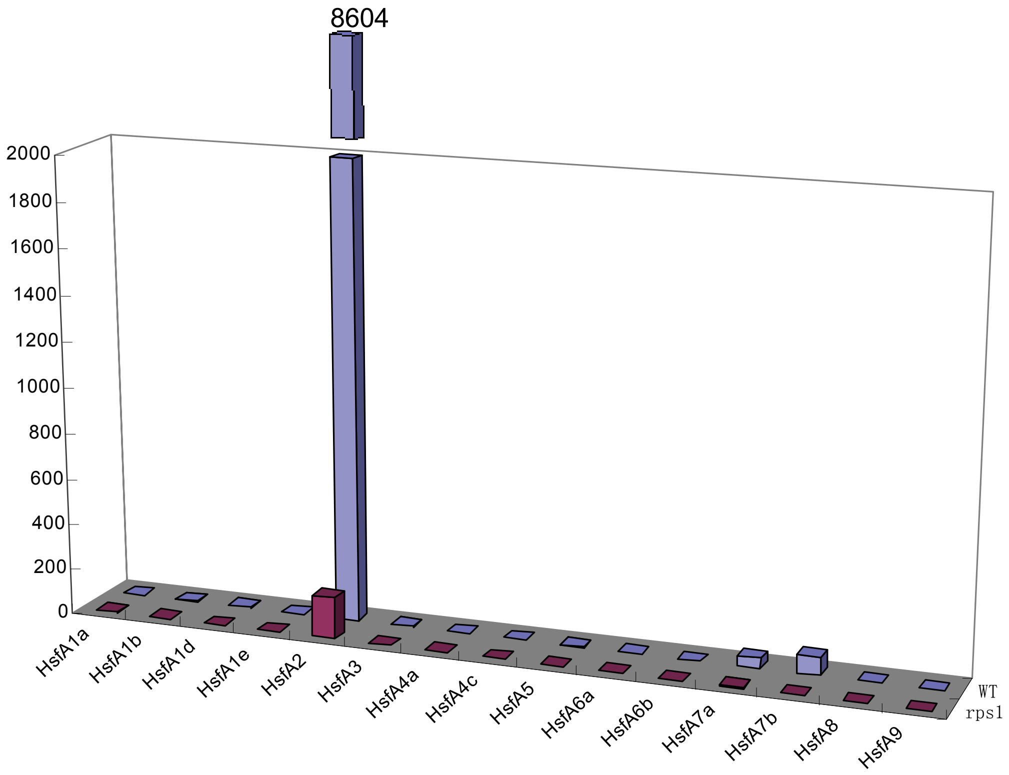

Figure S10. Heat-responsive expression analysis of HSF members in class A in wild type and *rps1* mutant plants.

qRT-PCR analysis of mRNA levels of 15 class A HSF members in detached, fully-extended WT and *rps1* leaves challenged with heat treatment (38°C) for 1 h in dark. *Actin2* was used as the internal standard. Error bars indicate standard deviations of three technical replicates, and the results were consistent in three biological replicates.
