# Supplementary material for: Downregulation of Chloroplast RPS1 Negatively Modulates Nuclear Heat-Responsive Expression of HsfA2 and Its Target Genes in Arabidopsis
Source: PLoS Genet. 2012 May 3;8(5):e1002669. doi: 10.1371/journal.pgen.1002669 (PMC3342936; doi:10.1371/journal.pgen.1002669)
Supplement: Figure S11 — Chloroplast localization patterns of RPS1-GFP in guard cells of epidermal peels. Chloroplast localization patterns were inspected with a confocal microscope. Epidermal peels were excised from the transgenic plants expressing the indicated RPS1-GFP proteins. (PDF) [file pgen.1002669.s011.pdf]

Figure S11. Yu et al.

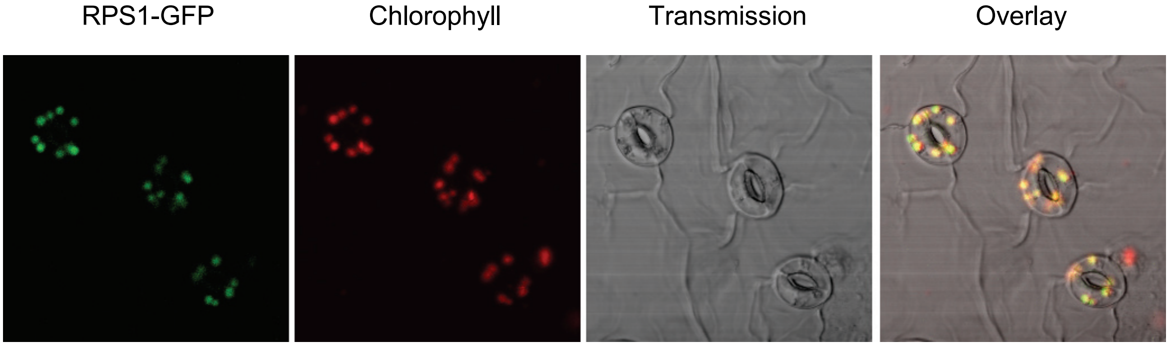

Figure S11. Chloroplast localization patterns of RPS1-GFP in guard cells of epidermal peels.

Chloroplast localization patterns were inspected with a confocal microscope. Epidermal peels were excised from the transgenic plants expressing the indicated RPS1-GFP proteins.
