# Supplementary material for: Downregulation of Chloroplast RPS1 Negatively Modulates Nuclear Heat-Responsive Expression of HsfA2 and Its Target Genes in Arabidopsis
Source: PLoS Genet. 2012 May 3;8(5):e1002669. doi: 10.1371/journal.pgen.1002669 (PMC3342936; doi:10.1371/journal.pgen.1002669)
Supplement: Figure S12 — Phylogenetic relationships of RPS1s in Arabidopsis thaliana (At5g30510), Spinacia oleracea (accession number: M82923), Synechocystis sp. PCC 6803 (accession number: gi|1652650), Marchantia polymorpha (accession number: gi|786212), Chlamydophia felis Fe/C-56 (accession number: AJ585191) and Plasmodium chabaudi (accession number: gi|70945988). A rooted phylogenetic tree was constructed using TreeView version 1.6.6 software with the neighbor-joining method based on ClustalW multiple alignments of the possible RPS1s. Bar = 0.1 amino acid substitutions per site. (PDF) [file pgen.1002669.s012.pdf]

Figure S12. Yu et al.

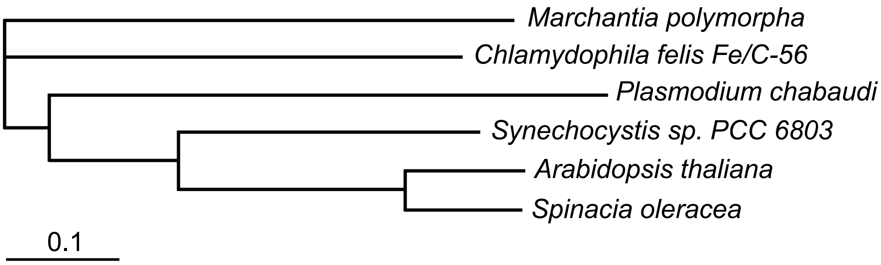

Figure S12. Phylogenetic relationships of RPS1s in *Arabidopsis thaliana* (At5g30510), *Spinacia oleracea* (accession number: M82923), *Synechocystis sp. PCC 6803* (accession number: gi|1652650), *Marchantia polymorpha* (accession number: gi|786212), *Chlamydophia felis Fe/C-56* (accession number: AJ585191) and *Plasmodium chabaudi* (accession number: gi|70945988).

A rooted phylogenetic tree was constructed using TreeView version 1.6.6 software with the neighbor-joining method based on ClustalW multiple alignments of the possible RPS1s. Bar = 0.1 amino acid substitutions per site.
