# Supplementary material for: Downregulation of Chloroplast RPS1 Negatively Modulates Nuclear Heat-Responsive Expression of HsfA2 and Its Target Genes in Arabidopsis
Source: PLoS Genet. 2012 May 3;8(5):e1002669. doi: 10.1371/journal.pgen.1002669 (PMC3342936; doi:10.1371/journal.pgen.1002669)
Supplement: Figure S13 — Knockdown of RPS17 expression in rps17 mutant plants leads to heat susceptibility. (A) Schematic diagram of RPS17 gene (At1g79850) showing the T-DNA insertion site. Open box indicates 5′or 3′UTR; Closed box indicates ORF. The T-DNA insertion site and positions of the start and stop codons are indicated (SALK_066943). (B) RPS17 mRNA levels in leaves of wild type and rps17 mutant plants were analyzed by qRT-PCR. Actin2 was used as the internal standard. (C) Western blot analysis of thylakoid membrane proteins extracted from WT and rps17 leaves. Equal protein loading was determined by contents (2 µg) of chlorophyll in thylakoid membrane extracts according to (Peng et al., 2006). (D) to (E) Heat-challenged phenotypes of wild type and rps17 mutant as examined with detached leaf (D) and whole plant (E) assays performed as described in Methods. (F) qRT-PCR analysis of mRNA levels of HsfA2 in detached, fully-extended WT and rps1 leaves challenged with heat treatment (38°C) for the indicated time in dark. For qRT-PCR analysis, Actin2 was used as the internal standard. Error bars indicate standard deviations of three technical replicates, and the results were consistent in three biological replicates. (PDF) [file pgen.1002669.s013.pdf]

Figure S13. Yu et al.

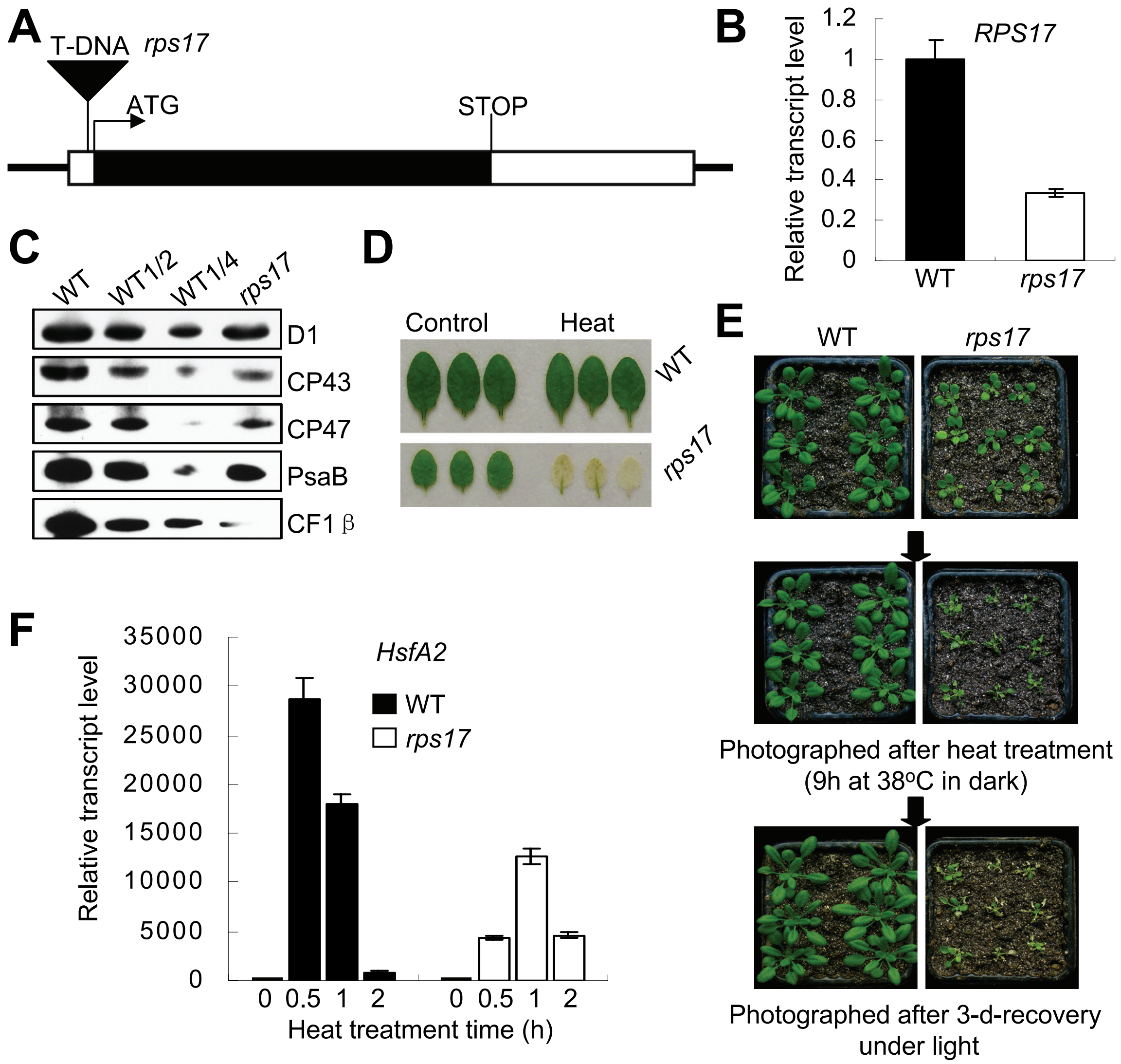

Figure S13. Knockdown of *RPS17* expression in *rps17* mutant plants leads to heat susceptibility.

(A) Schematic diagram of *RPS17* gene (At1g79850) showing the T-DNA insertion site. Open box indicates 5' or 3' UTR; Closed box indicates ORF. The T-DNA insertion site and positions of the start and stop codons are indicated (SALK_066943).
(B) *RPS17* mRNA levels in leaves of wild type and *rps17* mutant plants were analyzed by qRT-PCR. *Actin2* was used as the internal standard.
(C) Western blot analysis of thylakoid membrane proteins extracted from WT and *rps17* leaves. Equal protein loading was determined by contents (2 μg) of chlorophyll in thylakoid membrane extracts according to (Peng et al., 2006).
(D) to (E) Heat-challenged phenotypes of wild type and *rps17* mutant as examined with detached leaf (D) and whole plant (E) assays performed as described in Methods.
(F) qRT-PCR analysis of mRNA levels of *HsfA2* in detached, fully-extended WT and *rps1* leaves challenged with heat treatment (38°C) for the indicated time in dark.
For qRT-PCR analysis, *Actin2* was used as the internal standard. Error bars indicate standard deviations of three technical replicates, and the results were consistent in three biological replicates.
