# Supplementary material for: Downregulation of Chloroplast RPS1 Negatively Modulates Nuclear Heat-Responsive Expression of HsfA2 and Its Target Genes in Arabidopsis
Source: PLoS Genet. 2012 May 3;8(5):e1002669. doi: 10.1371/journal.pgen.1002669 (PMC3342936; doi:10.1371/journal.pgen.1002669)
Supplement: Figure S14 — Heat-responsive expression of HsfA2 is severely inhibited in wild type seedlings by Lincomycin treatment. qRT-PCR analysis of mRNA levels of HsfA2 in 6-d-old seedlings of WT challenged with heat treatment (38°C) for 2 h in dark under control or lincomycin treatment condition. Actin2 was used as the internal standard. Error bars indicate standard deviations of three technical replicates, and the results were consistent in three biological replicates. (PDF) [file pgen.1002669.s014.pdf]

Figure S14. Yu et al.

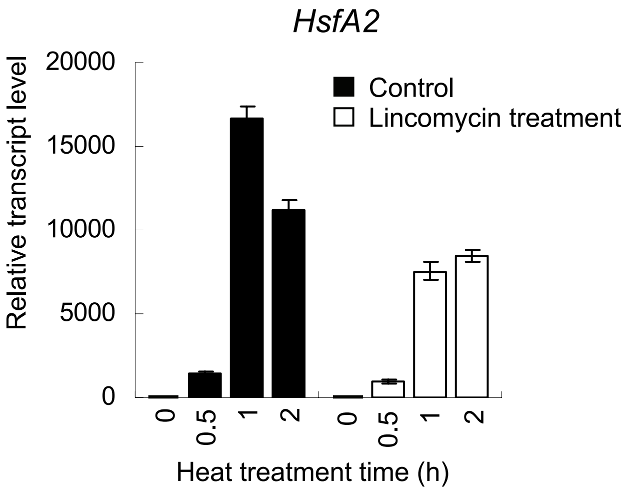

Figure S14. Heat-responsive expression of *HsfA2* is severely inhibited in wild type seedlings by Lincomycin treatment.

qRT-PCR analysis of mRNA levels of *HsfA2* in 6-d-old seedlings of WT challenged with heat treatment (38°C) for 2 h in dark under control or lincomycin treatment condition. *Actin2* was used as the internal standard. Error bars indicate standard deviations of three technical replicates, and the results were consistent in three biological replicates.
