# Supplementary material for: Downregulation of Chloroplast RPS1 Negatively Modulates Nuclear Heat-Responsive Expression of HsfA2 and Its Target Genes in Arabidopsis
Source: PLoS Genet. 2012 May 3;8(5):e1002669. doi: 10.1371/journal.pgen.1002669 (PMC3342936; doi:10.1371/journal.pgen.1002669)
Supplement: Table S1 — Sequences of the primers for constructs. (PDF) [file pgen.1002669.s015.pdf]

Supplemental Table S1. Sequences of the primers for constructs

Primers for constructs	
Primer Name	Sequence(5'-3')
ROX+	AAAGGATCCATGGCGTCTTTGGCTCAGCAATTC
ROX-	AAAGGTACCCTAAATATCAACTGCAGAAGGAATG
RGFP+	AAACTCGAGATGGCGTCTTTGGCTCAGCAATTC
RGFP-	AAAACTAGTGGAATATCAACTGCAGAAGGAATGTC
RComp+	AAAGGTACCGTCTCCGACCTATTATGACGAAC
RComp-	AGGTCTAGATCTGAAGATATCCATACCCAACAC
FRNAi+	AAACCCGGGCTCGAGCTGTGTGAGTGAGTGAGACTC
FRNAi -	CGCTCTAGATGACAAACTCTTCCACCATAC
RRNAi+	AAAGAGCTCCTCGAGCTGTGTGAGTGAGTGAGACTC
RRNAi-	CACGCGGCCGCTGACAAACTCTTCCACCATAC
121A2-F	AATCTAGACTCTGAGCTTATGGATTTGAG
121A2-R	AAGAGCTCGACCGCAACAAGTAGATGTG
RGUS+	AAAAAGCTTGTCTCCGACCTATTATGACGAAC
RGUS-	AGAGGATCCATAAGCATCTTCGAACATCTTCTTC
