# Supplementary material for: Downregulation of Chloroplast RPS1 Negatively Modulates Nuclear Heat-Responsive Expression of HsfA2 and Its Target Genes in Arabidopsis
Source: PLoS Genet. 2012 May 3;8(5):e1002669. doi: 10.1371/journal.pgen.1002669 (PMC3342936; doi:10.1371/journal.pgen.1002669)
Supplement: Table S2 — Sequences of the primers for qRT–PCR and RT–PCR. (PDF) [file pgen.1002669.s016.pdf]

Supplemental Table S2. Sequences of the primers for qRT-PCR and RT-PCR

Primers for qRT-PCR	
Primer Name	Sequence(5'-3')
RPS1-F	AACTGTTCTTCAGCCTGGTGACAC
PRS1-R	CTGTCTGAATGTCTGAGCCATCTC
HsfA2-F	TCGTCAGCTCAATACTTATGGATTC
HsfA2-R	CACATGACATCCCAGATCCTTGC
Hsp101-F	GAGGAGTTGCTTTGGCAGTC
Hsp101-R	CAGCGCCTGCATCTATGTAA
Hsp17.7-F	TCAGGTCCAGATAGAGAACGAGAAC
Hsp17.7-R	CCTCTCCATCCTCACAAACTTCAC
Hsp18.1-F	GGTTACCGGAGAATGCAAAGATG
Hsp18.1-R	CGATGGACTTGACTTGAGGCTTC
Hsp25.3-F	ACGGAGTCCTCTTTATCACTATCCC
Hsp25.3-R	GATCGAGTCCTACTGAATCTGG
Hsp70-F	GCAGAAGATTGAGAAGGCGATTG
Hsp70-R	CGCTCTAATCCACCTCTTCGATC
Gosl1-F	AGCCGTTCATCACCGCTCTTAC
Gosl1-R	ACTCCTGGCAACATTCAAGCAG
APX2-F	AATATGCTGCAGATGAGGATGC
APX2-R	CAAGAATCAAGGAGGTAGGAGATG
RPS17-F	ATGATAACGTCGTCCCTAAC
RPS17-R	CTCTTTTCCAGTCTGACCACG
Actin2-F	GGCAAGTCATCACGATTGG
Actin2-R	CAGCTTCCATTCCCACAAAC
Primers for RT-PCR	
Primer Name	Sequence(5'-3')
RPS1+	TGTAGCAGATAGCCAAGCTCAG
RPS1-	CTAAATATCAACTGCAGAAGGAATG
HsfA2+	ATGGAAGAACTGAAAGTGGAAATG
HsfA2-	GATCAATCTTTCTGAATCCATAAG
